# Sustainable Mizoroki–Heck Cross-Coupling Using a Pd(II)-Polymer as Precatalyst in 1‑Butanol

**DOI:** 10.1021/acsomega.6c01810

**Published:** 2026-06-15

**Authors:** Elvis Naoto Nishida, Laíze Zaramello, Mateus H. Keller, Bruno Luz da Silva, Raphaell Moreira, Thorsten M. Gesing, Bruno S. Souza

**Affiliations:** † Department of Chemistry, 28117 Federal University of Santa Catarina, Florianópolis, Santa Catarina 88040-900, Brazil; ‡ Universität Bremen, Institute of Applied and Physical Chemistry, Leobener Str. 6, Bremen D-28359, Germany; § Universität Bremen, Institute of Inorganic Chemistry and Crystallography, Leobener Str. 7, Bremen D-28359, Germany; ∥ Universität Bremen, MAPEX Center for Materials and Processes, Bibliothekstr. 1, Bremen D-28359, Germany

## Abstract

This work describes the application of a palladium-containing polymeric precatalyst (Pd/PECIm) for the sustainable Mizoroki–Heck (MH) cross-coupling reaction, focusing on the use of green solvents. Building on previous research in which Pd/PECIm was successfully applied to the Suzuki–Miyaura reaction, the cross-coupling between iodobenzene and ethyl acrylate was initially performed in water and 2-propanol (*i*PrOH). While low yields were obtained in water or *i*PrOH alone, good yields were obtained in a mixture of both solvents. However, partial polymer degradation was observed, which led to the formation of inactive “palladium black” aggregates, hindering catalyst reuse. The use of 1-butanol (1-BuOH) provided superior efficiency, and under optimized conditions, the system achieved 95% conversion to ethyl cinnamate in just 1.5 h with 0.5 mol% Pd. Imidization of the polymer matrix took place in 1-BuOH, which preserved its structural integrity and allowed for the controlled formation of stable Pd nanoparticles. The catalyst could be reused for at least five cycles. The methodology demonstrated a broad substrate scope, producing various cinnamate and stilbene derivatives with excellent yields, and was successfully scaled up 50-fold with a 93% yield. Overall, Pd/PECIm in 1-BuOH represents an efficient, stable, and environmentally friendly alternative for MH transformations.

## Graphical abstract

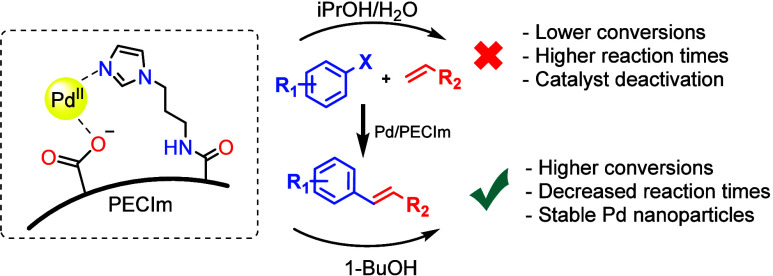

## 1 Introduction

Cross-coupling reactions catalyzed by palladium occupy a central position in the synthetic toolbox available for the preparation of small molecules, materials,^1–4^ and the development of novel bioorthogonal chemistry.^5,6^ Along with the Suzuki–Miyaura reaction, the Mizoroki–Heck (MH) reaction, initially proposed by Tsutomu Mizoroki^7,8^ and Richard F. Heck^9,10^ in the early 1970s, stands out as a popular Pd-catalyzed cross-coupling reaction. Originally, high amounts of Pd salts, such as Pd­(OAc)_2_, were employed, which generated the active Pd^0^ species in the presence of phosphines.

Following the 2010 Nobel Prize in Chemistry,^11,12^ the MH reaction continues to be widely studied due to its efficiency and versatility. However, the rising cost of palladium on the global market, as well as the depletion of its primary natural reserves, have raised significant concerns.^11,12^ In this context, the development of recyclable and more efficient palladium-based catalysts has become essential for creating more sustainable methodologies that are less harmful to both the environment and human health.^13,14^ These principles align with the United Nations Sustainable Development Goals (UN SDGs), particularly Goal 3 (Good Health and Well-Being), Goal 9 (Industry, Innovation, and Infrastructure), and Goal 12 (Responsible Consumption and Production).^15,16^ Consequently, collective efforts have been dedicated to the development of new palladium-based catalysts for the MH reaction. Notable approaches include the immobilization of palladium on polymer matrices,^17–19^ covalent organic frameworks,^2,20^ metal organic frameworks,^21,22^ and carbohydrate-derived supports.^23,24^ However, multiple synthetic steps are typically required for palladium immobilization onto heterogeneous matrices, often involving lengthy procedures, the use of toxic organic solvents, lengthy reactions, and the need for oxygen-free conditions.^25–33^

Recently, our research group has developed a palladium-containing polymeric material with imidazole ligands (Pd/PECIm), which has been successfully applied in the Suzuki–Miyaura reaction under environmentally benign conditions, including the use of green solvents (*i*PrOH/H_2_O mixture), mild temperatures, and the presence of oxygen.^19^ It was demonstrated that Pd/PECIm acts as a reservoir of Pd, which, once detached into the reaction media, catalyzes the formation of biphenyls.^28,34^ The presence of dissolved oxygen played a crucial role in regenerating Pd­(II),^35,36^ thereby preventing catalyst deactivation through irreversible Pd(0) aggregation and allowing catalyst reuse over 12 consecutive cycles. On the basis of these findings, herein we explore the potential application of Pd/PECIm in the MH reaction in benign solvents, contributing to the advance of more environmentally friendly synthetic methodologies.

## 2 Materials and Methods

### 2.1 Preparation of PECIm

Preparation of PECIm was performed as described previously by Leopoldino and co-workers.^37^ Poly­(ethylene-*alt*-maleic anhydride) (PEMA) (3.00 g, 23.8 mmol; average Mw 100,000–500,000, monomer molecular weight = 126 g mol^–1^) was dissolved in 75 mL of acetone at 25 °C in a 250 mL round-bottom flask under magnetic stirring. After stirring for 30 min, a solution of 1-(3-aminopropyl)-imidazole (APIm; 3.4 mL, 28.5 mmol, 1.2 equiv) in 25 mL of acetone was added dropwise. The solution turned cloudy upon the first drop of APIm, indicating an immediate reaction. The reaction was continued for 24 h, then the polymer was collected by centrifugation and washed several times with acetone. The white solid obtained (5.42 g) was stored in a desiccator overnight, followed by drying in a vacuum oven at 60 °C for 8 h.

### 2.2 Preparation of Pd/PECIm

The preparation of Pd/PECIm was performed as described previously by Nishida and co-workers.^19^ In an Erlenmeyer flask, 100 mL of a 0.2 mol L^–1^ aqueous solution of PECIm (0.502 g; MM_monomer_ = 251.17 g mol^–1^) was freshly prepared. The resulting solution was magnetically stirred until the complete solubilization of the polymer. To this, 100 mL of an aqueous solution containing 0.326 g of K_2_PdCl_4_ (0.1 mol L^–1^) was added dropwise with the aid of an addition funnel under strong magnetic stirring. After the addition of a few drops, formation of small pale-yellow precipitates was observed, indicating the formation of the metal–polymer complex. After the complete addition of K_2_PdCl_4_ to the system, it was left stirring overnight. The resulting precipitate was filtered, washed with deionized water (4 × 100 mL), acetone (2 × 100 mL), and dried in a vacuum oven at 60 °C for 6 h. The pale yellow solid (0.575 g) was obtained in about 70% yield with respect to Pd­(II) from the starting salt and has 11.3 wt% Pd as determined by FAAS analysis. Elemental analysis gives (%) C 41.36; H 5.29; N 9.86; O 32.09, indicating a 1:2 Pd-to-imidazole ratio.

### 2.3 Optimization of the MH Reaction between iodobenzene and Ethyl Acrylate

Optimization experiments were carried out in 2 mL glass ampules. Iodobenzene (0.5 mmol), ethyl acrylate (1.0 mmol), triethylamine (1.0 mmol), and 1.0 mL of solvent (*i*PrOH, *i*PrOH:H_2_O (1:1 v/v), ethanol, or 1-BuOH) were introduced into the ampule. Finally, Pd/PECIm was added to the system. The ampule was sealed using a torch and immersed in a preheated oil bath under vigorous magnetic stirring for a specified reaction time. GC-MS analysis was used to monitor product formation. Reactions were allowed to run for a maximum of 24 h. After the desired time, the reaction mixture was extracted with ethyl acetate (4 × 1 mL), and the organic phase was washed with brine (2 × 1 mL) and water (4 × 1 mL). After drying over anhydrous Na_2_SO_4_, the solvent was removed under reduced pressure. An aliquot of the resulting solution was diluted in HPLC-grade hexane and analyzed by GC/MS. Quantification was performed using calibration curves constructed for iodobenzene and ethyl cinnamate. In all cases, yield and conversion converged within 5% error.

### 2.4 General Procedure for MH Reaction

Except for the use of 8 mL screw-cap glass tubes instead of glass ampules and the use of 1-BuOH only, a procedure similar to that of Section 2.3 was employed. After phase separation, which could be accelerated by centrifugation, the organic phase was dried with sodium sulfate (Na_2_SO_4_) and filtered, and the solvent was removed under vacuum. The obtained solid/oil was analyzed by ^1^H and ^13^C NMR (NMR spectra available in the Electronic Supporting Information (ESI)), allowing the determination of the reaction yield. *Trans*-4-Acetylstilbeneobtained from styrene and 4-iodoacetophenone (entry 6 of Table 2) and *trans*-4-styrylpyridine (entry 16 of Table 2) from iodobenzene and 4-vinylpyridine were purified by column chromatography, whereas the remaining products were obtained in satisfactory purity and did not require further purification.

### 2.5 Kinetics of Ethyl Cinnamate Formation

In a 4 mL GC/MS vial, iodobenzene (1.0 mmol) and 2.0 mL of solvent (1-BuOH or *i*PrOH/H_2_O) were added. Subsequently, the alkene (2.0 mmol) was introduced, and the reaction mixture was stirred for 1 min. Finally, Pd/PECIm (5.0 mg, 0.52 mol% Pd) was added to the vial. The reaction was carried out at 95 °C. Aliquots were withdrawn at various reaction times, diluted 10,000-fold with ethanol 99.5% ACS, and analyzed by UV–Vis spectroscopy at the maximum absorption wavelength of ethyl cinnamate (λ_max_ = 276 nm).

### 2.6 Recycling Experiments in MH Reaction

The reaction was carried out similarly to the procedure described in Section 2.5. An aliquot of the reaction media was collected at *t* = 90 min, diluted 10,000-fold with 99.5% ethanol (ACS grade), and analyzed by UV–Vis spectroscopy at the maximum absorption wavelength of ethyl cinnamate (λ_max_ = 276 nm). After analysis, the supernatant was separated by centrifugation (3000 rpm, 5 min), and the catalyst was washed with the same solvent used in the MH reaction (15 mL), vortexed (2000 rpm, 1 min), and centrifuged again (3000 rpm, 5 min). This washing procedure was repeated four times. The recovered catalyst was then transferred back to the same vial used in the first cycle and recharged with the reactants, base, and solvent.

### 2.7 FTIR Analysis

After the MH reaction, Pd/PECIm was recovered by centrifugation and washed with acetone (2 × 5 mL). The samples were left to dry at room temperature (25 °C) and placed under vacuum to remove the solvent. KBr pellets were prepared and the scan was recorded in the 4000–400 cm^–1^ range.

### 2.8 Powder X-Ray Diffraction (XRPD) Analysis

XRPD data collections were carried out on a Bruker D8 Advance diffractometer using Ge(111)-monochromatized CuKα1 radiation (λ_Kα1_ = 154.05929(5) pm) in Bragg–Brentano geometry and a LynxEye-XE detector. These data were collected at ambient conditions from 5° to 135° 2θ with a step width of 0.0147° 2θ and a measurement time of 484 s per step. The fundamental parameter approach, where the fundamental parameters were fitted against LaB_6_ standard material, was applied for the Pawley fits as well as the Rietveld refinements using the “DiffracPlus Topas 4” software (Bruker AXS GmbH, Karlsruhe, Germany). Comprehensive information regarding the determination of average crystallite size and size distribution, the fitting procedure, and additional information are given in the ESI.

### 2.9 X-Ray Photoelectron Spectroscopy (XPS) Analysis

Samples were mounted on nonconductive double-sided adhesive tape (3 M Scotch Tape). The XPS analyses were carried out with a Kratos AXIS Supra X-ray photoelectron spectrometer using a monochromatic Al K­(alpha) source (15 mA, 15 kV). The instrument work function was calibrated to give a binding energy (BE) of 83.96 eV for the Au 4f7/2 line for metallic gold, and the spectrometer dispersion was adjusted to give a BE of 932.62 eV for the Cu 2p_3/2_ line of metallic copper. The Kratos charge neutralizer system was used on all specimens. Survey scan analyses were carried out with an analysis area of 300 × 700 μm and a pass energy of 160 eV. High-resolution analyses were carried out with an analysis area of 300 × 700 μm and a pass energy of 20 eV. Spectra were charge-corrected to the main line of the carbon 1s spectrum (adventitious carbon or aliphatic carbon) set at 284.8 eV. CasaXPS software (version 2.3.26) was used in the analysis.

## 3 Results and Discussion

### 3.1 MH Reaction Optimization

The MH reaction between iodobenzene and ethyl acrylate was selected to evaluate the catalytic performance of Pd/PECIm under varying conditions of solvent, reaction time, and catalyst loading (Table 1). Guided by the solvent selection principles outlined by Prat et al.,^38^ isopropanol (*i*PrOH), classified as a recommended solvent, was initially employed. The reaction was conducted at 80 °C (near its boiling point), with 0.5 mol % Pd relative to iodobenzene for 24 h, affording only a 17% yield (entry 1). As previously observed in the SM cross-coupling using Pd/PECIm, the presence of water promotes the release of Pd from the polymer.^19^ Indeed, when a 1:1 mixture of *i*PrOH/H_2_O was used under identical conditions, a significant increase in ethyl cinnamate formation was obtained compared to *i*PrOH alone (entry 2). A similar yield was achieved in pure water after 24 h (entry 3). However, under this condition, the final solution was homogeneous, suggesting polymer degradation by hydrolysis, thus hindering catalyst recovery.

**Table 1:**
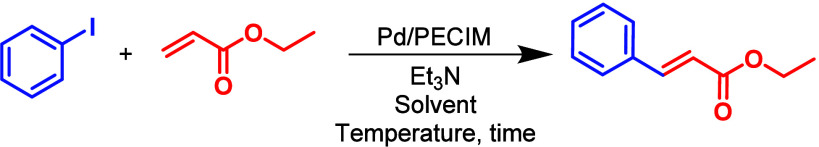
Optimization of Reaction Conditions for MH Reaction between iodobenzene and Ethyl Acrylate Catalyzed by Pd/PECIm^a^

**entry**	Pd/mol %	*T*/°C	*t*/h	**solvent**	yield/%^b^
1	0.5	80	24	*i*PrOH	17
2	0.5	80	24	*i*PrOH/H_2_O	66
3	0.5	80	24	H_2_O	67
4	1.0	80	24	*i*PrOH/H_2_O	69
5	1.5	80	24	*i*PrOH/H_2_O	70
6	2.0	80	24	*i*PrOH/H_2_O	69
7	0.5	90	24	*i*PrOH/H_2_O	88
8	1.0	90	24	*i*PrOH/H_2_O	87
9	0.5	80	24	EtOH	79
10	0.5	95	1.5	1-BuOH	95
11^c^	-	95	1.5	1-BuOH	0
12^d^	-	95	1.5	1-BuOH	0

a Reaction conditions: iodobenzene (0.5 mmol), ethyl acrylate (1.0 mmol), Et_3_N (1.0 mmol), solvent (1.0 mL), and Pd/PECIm (ranging from 2.5 to 10 mg).

b Yield determined by GC/MS with a calibration curve for ethyl cinnamate.

c A total of 2.5 mg of PECIm was used.

d No additional components were added.

To probe the effect of precatalyst loading, reactions were performed in *i*PrOH/H_2_O (1:1). Surprisingly, increasing Pd/PECIm up to 2 mol% did not improve yields (entries 4–6). This outcome is consistent with the findings by Mori and co-workers^7^ and may be related to the limited solubility of Pd complexes in the medium. Thus, despite higher catalyst amounts, most of the Pd likely remained out of phase and unavailable for catalysis. Since increasing loading did not enhance product formation, the temperature was raised to 90 °C, which delivered a 20% yield improvement (entry 7). As at 80 °C, raising Pd/PECIm to 1.0 mol% at 90 °C did not further increase yield (entry 8). Interestingly, at 0.5 mol% and 80 °C, ethanol exhibited higher efficiency than the *i*PrOH/H_2_O solvent system (entry 9). However, further temperature increases in ethanol would result in pressure buildup within the sealed glass ampules, thereby elevating the associated safety risks.

Typically, MH coupling reactions are performed at temperatures between 80 and 140 °C. To enable safe operation above 90 °C, 1-BuOH was selected due to its boiling point (120 °C) and its classification as a recommended solvent in the literature.^38–40^ Under these conditions, the use of 1-BuOH led to a remarkable improvement in ethyl cinnamate formation, achieving 95% conversion at 95 °C after only 1.5 h (entry 10). Notably, at the end of the reaction, the polymer exhibited a more consistent solid character, in contrast to other conditions where a gelatinous material was observed postreaction, likely due to the partial decomposition of PECIm (as discussed later). Finally, control experiments performed under the optimized conditions in 1-BuOH in the absence of Pd, either with or without PECIm (entries 11 and 12), led to no detectable product formation, confirming the essential role of the palladium catalyst.

The use of 1-BuOH as a reaction solvent in the MH reaction is uncommon, with only a few precedents reported by Yamada et al.^41^ and Gole et al.^42^ In the study by Yamada, Pd nanoparticles (NPs, 0.3 mol%) supported on silica nanowires (SiNA–Pd) afforded good conversions but required a 24 h reaction time, likely due to the use of Pd(0). Moreover, tetrabutylammonium acetate (TBAA) was necessary to promote the leaching and stabilization of Pd NPs in solution.^41^ Similarly, Gole employed Pd NPs supported on MOFs with metal loadings up to 27 wt%. Despite their high dispersibility, these systems also required prolonged reaction times (24 h) and the addition of TBAA.^42^ In comparison, Pd/PECIm presents several advantages, including a reduced reaction time, no requirement for additives, and a simpler preparation process. To get detailed information regarding the use of Pd/PECIm in *i*PrOH/H_2_O (1:1) and 1-BuOH, kinetic experiments and potential catalyst recycling were analyzed.

### 3.2 Kinetic and Recycling Experiments for Pd/PECIm in MH Reaction

Figure 1A,B shows the kinetics for ethyl cinnamate formation in an *i*PrOH/H_2_O mixture and the corresponding recycling results. As can be observed, the maximum reaction yield is reached between 16 and 24 h at 80 °C. Therefore, recycling studies in *i*PrOH/H_2_O were carried out after 24 h. In the first cycle, the material was highly active, affording approximately 80% conversion to the desired product. The slight decrease compared to the sealed ampule experiment (entry 7 of Table 1) is attributed to substrate volatilization, as the reaction flask has a larger headspace volume. After 24 h, the product was extracted with EtOAc, and the aqueous Pd/PECIm dispersion was recharged with base and substrates to initiate a new catalytic cycle. The second, third, and fourth cycles afforded 70%, 60%, and 60% yields, respectively (Figure 1B).

**Figure 1:**
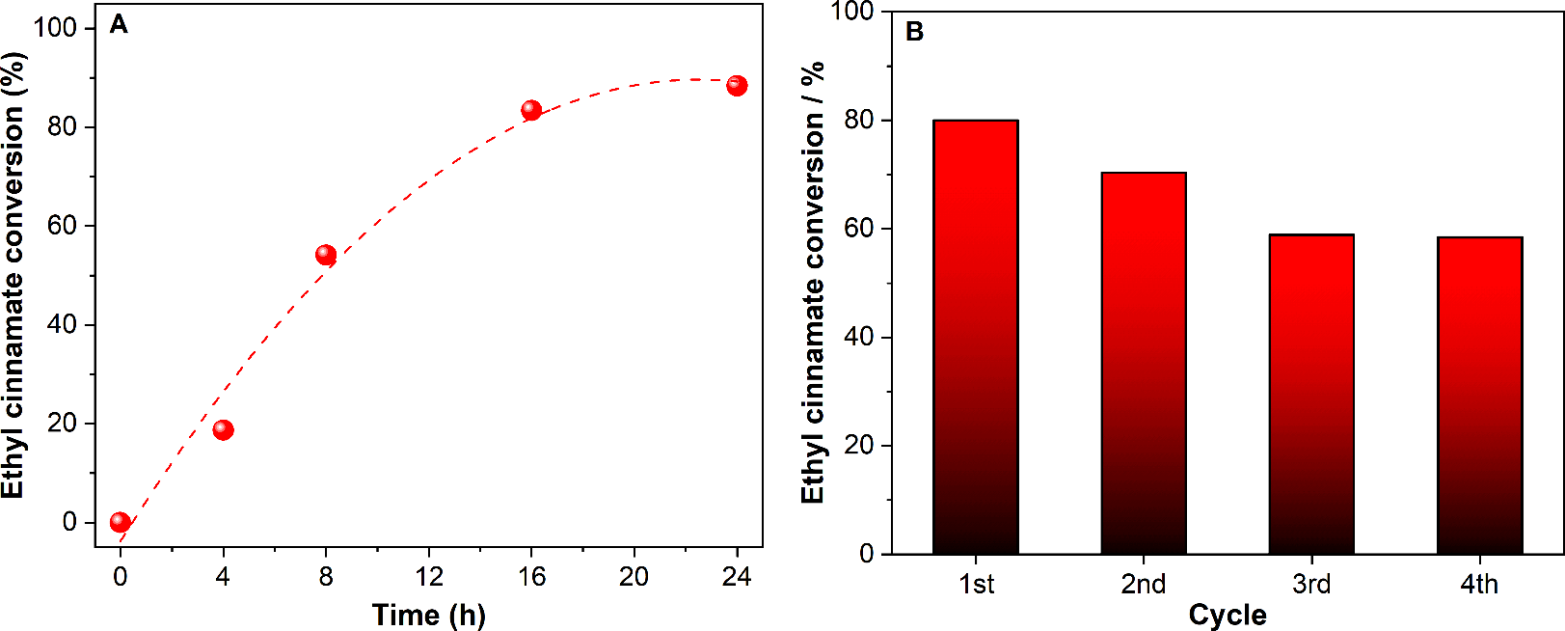
(A) Kinetic profile and (B) recycling of Pd/PECIm in the Mizoroki–Heck reaction. Kinetic conditions: iodobenzene (1.0 mmol), ethyl acrylate (2.0 mmol), Et_3_N (2.0 mmol), *i*PrOH (1.0 mL), H_2_O (1.0 mL), Pd/PECIm (0.52 mol% Pd, 5.0 mg), 80 °C. The recycling reactions were conducted on a 2-fold scale for 24 h.

From the second cycle onward, the color of Pd/PECIm became darker, indicating the formation of Pd NPs or submicrometric aggregates. In situ reduction of Pd­(II) to Pd(0) can occur via triethylamine oxidation or the Wacker-type reaction with alkenes. Once formed, Pd(0) tends to aggregate, as reported, for example, by De Vries and co-workers.^43–45^ Another explanation for the ease of Pd NP formation in MH reactions was recently proposed by Polynski and Ananikov,^46^ who studied the formation of Pd NPs through density functional theory (DFT) calculations. Their results indicate that Pd–Pd bonds are energetically weaker than Pd–*X* bonds (where *X* = Cl or Br), favoring leaching during the oxidative addition step. Additionally, the cohesion energy of Pd species depends on the cluster size, with Pd_4_ having a cohesion energy of −38 kcal mol^–1^ atom^–1^, while Pd_79_ and Pd_140_ had cohesion energies of −69.1 and −72.2 kcal mol^–1^ atom^–1^, respectively. This behavior further promotes the aggregation of in situ-generated Pd(0) species into larger particles, ultimately leading to a progressive decline in catalytic activity.

A direct correlation between the decrease in catalytic activity and the increasing presence of Pd black was confirmed by TEM analysis of the catalyst recovered after the first and fourth cycles. As shown in Figure S1, spherical particles of about 20–30 nm in diameter were observed in both samples. However, after the fourth cycle, aggregates exceeding 200 nm were also detected, indicating that the nanoparticles grew in a disordered manner, ultimately forming Pd black. This loss of activity is consistent with the detachment mechanism of the molecular Pd(0) species. After completing a catalytic cycle, these species are regenerated and can initiate new catalytic cycles. However, once Pd nanoparticles begin to form, they tend to aggregate and increase in size, thereby reducing the available active surface area. This aggregation leads to diminished catalytic efficiency over successive cycles.

The aforementioned results align with the proposed mechanistic role of Pd/PECIm in Suzuki–Miyaura reactions.^19^ In this system, the polymer acts as a reservoir that gradually releases trace amounts of Pd into the reaction medium. Among these species, Pd(0) participates in the catalytic cycle, while the polymer matrix inhibits the uncontrolled aggregation of palladium into relatively large and catalytically inactive palladium black. Given the high intrinsic activity of palladium, even trace levels are sufficient to efficiently promote the reaction.^47–49^ This so-called *boomerang mechanism*, in which the support primarily serves as a reservoir of a cocktail of Pd species, has been widely described in related systems.^50–52^

One possible reason for Pd aggregation is the partial decomposition of the polymer during the reaction in an *i*PrOH/H_2_O mixture, which reduces its ability to stabilize Pd NPs. To investigate this, FTIR spectra of the pristine and recovered catalysts were compared (Figure S2). Relative to the fresh Pd/PECIm sample, the recovered catalyst exhibited weaker signals associated with the carbonyl group of the amide moiety (1638–1526 cm^–1^) and a significant increase in the carboxylate asymmetric stretching band at 1649 cm^–1^. These spectral changes indicate partial degradation of the polymer side chains, probably through hydrolysis of the amide bond.

We then investigated the kinetics and recycling behavior of Pd/PECIm in 1-BuOH. UV–Vis monitoring of the first cycle revealed an induction period, corresponding to partial activation of the precatalyst into Pd(0) (Figure 2A). The second run proceeded more rapidly, consistent with the presence of active species already formed during the first cycle. Both runs were performed on the same day. In contrast, the third experiment, conducted the following day, resembled the kinetics of the first cycle, suggesting that reoxidation of Pd(0) to Pd­(II) had occurred overnight. Despite these variations, recycling experiments demonstrated excellent stability in 1-BuOH. Ethyl cinnamate yields consistently ranged from 90% to 100% across five consecutive cycles (Figure 2B), highlighting the robustness of the system under this condition.

**Figure 2:**
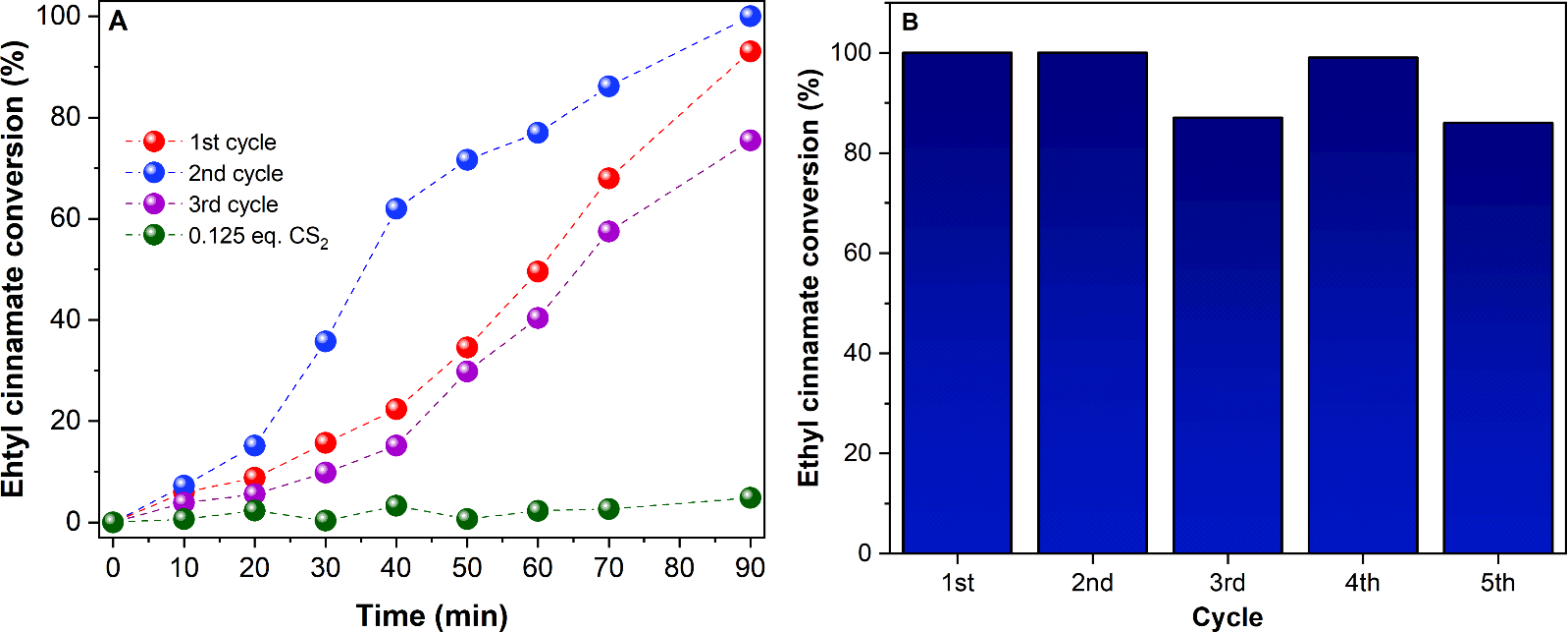
(A) Kinetic profile and (B) recycling of Pd/PECIm in the MH reaction. Kinetic conditions: iodobenzene (1.0 mmol), ethyl acrylate (2.0 mmol), Et_3_N (2.0 mmol), 1-BuOH (1.0 mL), Pd/PECIm (0.52 mol% Pd, 5.0 mg), 95 °C. The recycling reaction was performed on a 2-fold scale for 90 min.

As shown in Figure 2A, the addition of CS_2_ after 20 min completely halted the reaction, confirming that Pd(0) is the catalytically active species. Moreover, since only 0.12 equiv of CS_2_ relative to Pd was sufficient to fully inhibit the reaction, it can be inferred that only a fraction of the Pd present in the precatalyst is converted to the active species.

XPS analysis provided further evidence regarding the oxidation states of Pd. As shown in Figure S3, pristine Pd/PECIm displays signals centered at 338.0 and 343 eV, characteristic of Pd­(II).^19,53,54^ After the first MH catalytic cycle, additional signals at 341.0 and 336.0 eV, attributed to Pd(0), were observed. Together with the kinetic data, these results indicate that while some Pd is reduced to Pd(0) during the reaction, the majority remains in the +2 oxidation state in the recovered material.

The formation of Pd(0) NPs was also confirmed by XRPD analysis. After the first cycle, the average crystallite size determined by the Rietveld refinements was approximately 16 nm. Importantly, after the fifth cycle, the average size increased only slightly to 18 nm, demonstrating that the NP growth was effectively suppressed in 1-BuOH. TEM analysis of the catalyst recovered after the fifth cycle (Figure 3) revealed nearly spherical particles with diameters consistent with the XRPD data. Importantly, micrometric aggregates, similar to those observed in *i*PrOH/H_2_O, were absent, confirming that in 1-BuOH, the polymeric stabilizer successfully prevented particle agglomeration, maintaining the aggregates within the nanometric range.

**Figure 3:**
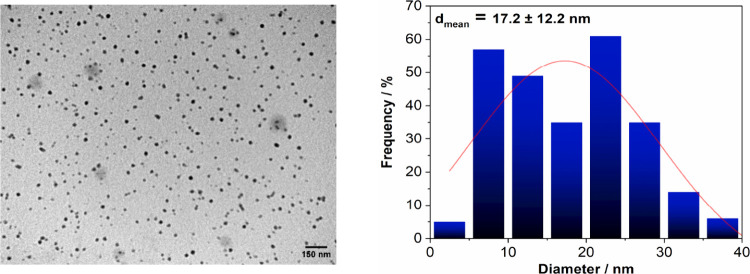
Micrographs obtained from TEM analysis of the catalyst used five times. Histograms obtained from the approximate counting of 250 particles.

The FTIR spectrum of the catalyst recovered after the fifth cycle is shown in Figure 4. The characteristic signals of the imidazole ring (819, 643–623 cm^–1^) remained unchanged, confirming that these groups are preserved during the reaction. Conversely, the amide bands disappeared, and a new absorption band was observed at 1700 cm^–1^. This can be attributed to the formation of a cyclic imide, arising from the reaction between vicinal amide and carboxylate groups at high temperature.^37^ Thus, although the polymer stabilizer undergoes structural modifications during the MH reaction, the imidazole moieties remain anchored to the polymer backbone and continue to stabilize the palladium species.

**Figure 4:**
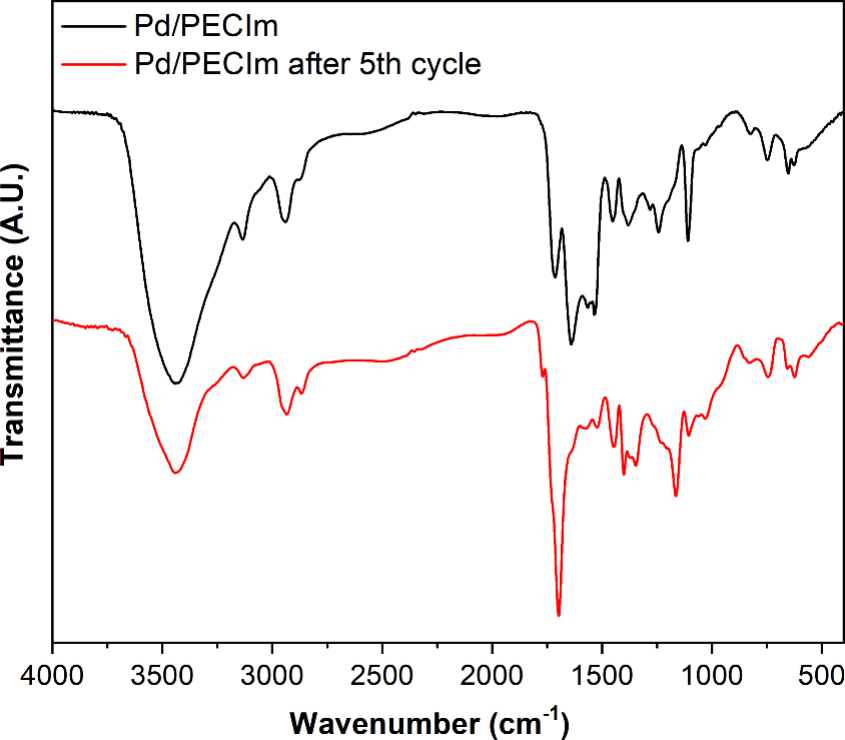
FTIR spectra of Pd/PECIm before use and after the fifth cycle in the MH reaction between iodobenzene and ethyl acrylate in 1-BuOH.

### 3.3 Substrate Scope for MH Coupling Reaction

After establishing the optimal reaction conditions and confirming the recyclability of Pd/PECIm in the coupling of iodobenzene with ethyl acrylate, the precatalyst was subsequently applied to a broader range of substrates to evaluate the influence of substituents on the MH reaction. The results are summarized in Table 2.

**Table 2:**
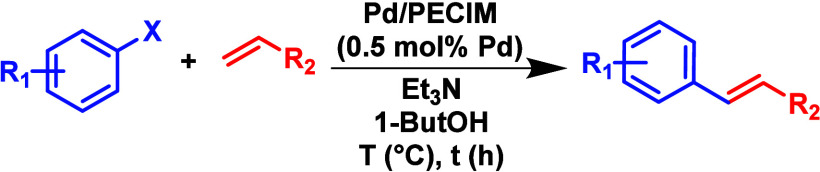
Scope of MH Coupling Reaction Catalyzed by Pd/PECIm^a^

**entry**	***X***	***R*** ^**1**^	***R*** ^**2**^	*t*/h	yield/%^b^	**TON**	**TOF/h** ^**–1**^ ^c^
1	I	H	CO_2_CH_3_	2	91	176	88
2	I	H	CO_2_CH_2_CH_3_	1.5	95	182	121
3	I	H	CO_2_(CH_3_)_3_	2	95	182	91
4^d^	I	H	C_6_H_5_	24	91	176	7
5^d^	I	4-CH_3_O	C_6_H_5_	24	73	141	6
6^d^	I	4-CH_3_CO	C_6_H_5_	48	66	127	3
7	I	4-CH_3_O	CO_2_CH_2_CH_3_	3	90	173	58
8	I	4-NO_2_	CO_2_CH_2_CH_3_	4	90	173	43
9	I	2-CH_3_	CO_2_CH_2_CH_3_	2	95	182	91
10	I	4-CH_3_O	CO_2_C(CH_3_)_3_	2	92	178	89
11	I	2-CH_3_	CO_2_C(CH_3_)_3_	2	91	176	88
12^d^	Br	H	CO_2_C(CH_3_)_3_	48	15	29	0.6
13^d^	Br	4-NO_2_	CO_2_CH_2_CH_3_	4	90	173	43
14^d^	Br	4-CH_3_CO	C_6_H_5_	48	56	108	2
15^d^	Cl	H	CO_2_C(CH_3_)_3_	56	0	0	0
16^d^	I	H	4-NC_5_H_5_	24	46	89	4
17^e^	I	H	CO_2_CH_2_CH_3_	2	93	172	86

a Reaction conditions: aryl halide (0.5 mmol), alkene (1.0 mmol), Et_3_N (1.0 mmol), *n*-BuOH (1.0 mL), Pd/PECIm (2.5 mg), 95 °C.

b Isolated yield.

c TOF was calculated based on the total amount of Pd atoms in Pd/PECIm.

d Performed at 120 °C.

e Performed on a 50× scale.

Excellent yields were obtained for the coupling of iodobenzene with methyl, ethyl, and *tert*-butyl acrylates (entries 1–3). In contrast, the use of styrene as the alkene substrate required a longer reaction time to afford *trans*-stilbene in 91% yield (entry 4). When compared to the less bulky and more reactive acrylic esters, this reduced efficiency can be attributed to the steric and electronic effects.

When styrene was coupled with an electron-rich aryl iodide (4-iodoanisole), the yield of the desired product decreased significantly to 73% (entry 5). Conversely, the use of an electron-withdrawing aryl iodide extended the reaction time to 48 h, ultimately affording the corresponding stilbene in 66% yield (entry 6).

By varying the electron-donating and -withdrawing groups on the aryl iodides, excellent yields of the desired coupling products were obtained. These reactions required only a slight increase in reaction time compared to those conducted with iodobenzene (entries 7 and 8).

To probe steric effects, 2-iodotoluene was employed as an aryl halide. The corresponding cinnamic ester was obtained in excellent yield (entry 9), indicating that the ortho-methyl substituent exerts minimal interference during the oxidative addition step. Similarly, aryl iodides bearing electron-donating groups reacted efficiently with *tert*-butyl acrylate, affording 92% yield with 4-iodoanisole and 91% with 2-iodotoluene (entries 10 and 11).

While chlorobenzene did not couple with *tert-*butyl acrylate even after being heated at 120 °C for 2 days, 15% yield was obtained from the reaction of bromobenzene (entry 12). Conversely, bromides bearing electron-withdrawing substituents provided good to excellent results (entries 13 and 14).

The cross-coupling involving iodobenzene and 4-vinylpyridine afforded *trans*-4-styrylpyridine in moderate yield (entry 16). The observed drop in yield compared to styrene is in agreement with the known limitations reported for the MH reactions involving vinylpyridines^55^ and can stem from the electron-withdrawing effect of the nitrogen as well as its ability to coordinate to Pd species.^56^

Finally, to evaluate the robustness of the proposed methodology, the reaction between iodobenzene and ethyl acrylate was scaled up 50-fold. Ethyl cinnamate was obtained in 93% yield (4.00 g), confirming that the methodology remains consistent with the small-scale results (entry 17).

## 4 Conclusions

In this work, a polymer-supported palladium precatalyst (Pd/PECIm) was successfully applied to the Mizoroki–Heck coupling reaction between various aryl halides and alkenes. Optimization studies demonstrated that the catalytic activity was strongly influenced by the reaction solvent, with 1-BuOH providing the highest conversion (95%) to ethyl cinnamate within only 1.5 h at 95 °C and 0.5 mol% Pd. This solvent not only enhanced the reaction efficiency but also preserved the structural integrity of the polymeric matrix through imidization, in contrast to the reactions carried out in *i*PrOH/H_2_O, where partial hydrolysis of the pendant groups, with concomitant formation of Pd black, was observed.

Kinetic and recycling studies revealed that Pd/PECIm can be efficiently reused for at least five consecutive cycles in 1-BuOH without a significant loss of catalytic activity. The good catalytic performance under these conditions was attributed to the controlled formation of Pd nanoparticles with an approximate size of 16 nm. The polymeric imidazole-based support (PECIm) played a dual role, acting both as a stabilizer and modulator of the Pd species, preventing excessive aggregation and maintaining catalytic efficiency.

The system exhibited a broad substrate scope, effectively catalyzing the coupling of various aryl halides with acrylates and styrene derivatives to afford the desired cinnamate and stilbene products in excellent yields. The methodology was also successfully scaled up 50-fold, maintaining high conversion and yield, thus demonstrating its practical applicability. Nonetheless, the approach remains limited to iodide and bromide derivatives and is less efficient for heterocyclic substrates.

Overall, Pd/PECIm represents a straightforward, robust, recyclable, and environmentally friendly catalytic system for the Mizoroki–Heck reaction, combining high activity, operational simplicity, and stability under mild and green reaction conditions. Its performance in 1-BuOH as a green solvent highlights the potential of polymer-supported palladium catalysts for sustainable organic transformations.

## Supplementary Material



## Notes

TEM micrographs of recovered catalyst employed in *i*PrOH/H_2_O; FTIR spectra of fresh Pd/PECIm, hydrolyzed PEMA and Pd/PECIm after the fourth reuse in iPrOH/H_2_O; XPS spectra of fresh and recovered Pd/PECIm after reaction in 1-BuOH; ^1^H NMR and ^13^C NMR of coupling products; UV–vis spectra and calibration curve for ethyl cinnamate; XRDP data refinement details (PDF)

## References

[1] Brown N., Zhang Q., Alsudairy Z., Dun C., Nailwal Y., Campbell A., Harrod C., Chen L., Williams S., Urban J. J., Liu Y., Li X. (2024). Mechanochemical
in
Situ Encapsulation of Palladium in Covalent Organic Frameworks. ACS Sustainable Chem. Eng..

[2] Li W.-J., Chen Y.-X., Kang S.-L., Mo L.-P., Zhang Z.-H. (2023). Design,
Synthesis and Characterization of Palladium-Functionalized Covalent
Organic Framework and Its Application as Heterogeneous Catalysis for
C–H Arylation of Azoles. Chem. A Eur.
J..

[3] Wan D., Che Z., Mo L., Hu M., Li J., Shi F., An Z., Li J. (2022). Synthesis and Properties of Fluorinated Terphenyl Liquid
Crystals Utilizing 5,6-Dihydro-4H-Cyclopenta­[b]­Thiophene as Core Unit. J. Mol. Struct..

[4] Liang Y., Gao T., Hu X., Liu N., Liu X., Gao H., Xiao Y. (2024). The Effect of Fluorination
on the Liquid Crystal and Optical Behaviors
of Amphiphilic Cyanostilbene-Based Mesogens. J. Mol. Struct..

[5] Forno G. M. D., Latocheski E., Navo C. D., Albuquerque B. L., John A. L. S., Avenier F., Jiménez-Osés G., Domingos J. B. (2024). Interplay of Chloride
Levels and Palladium­(II)-Catalyzed
O-Deallenylation Bioorthogonal Uncaging Reactions. Chem. Sci..

[6] Dal
Forno G. M., Latocheski E., Beatriz Machado A., Becher J., Dunsmore L., St. John A. L., Oliveira B. L., Navo C. D., Jiménez-Osés G., Fior R., Domingos J. B., Bernardes G. J. L. (2023). Expanding Transition Metal-Mediated
Bioorthogonal Decaging to Include C–C Bond Cleavage Reactions. J. Am. Chem. Soc..

[7] Mori K., Mizoroki T., Ozaki A. (1973). Arylation of Olefin with iodobenzene
Catalyzed by Palladium. BCSJ..

[8] Mizoroki T., Mori K., Ozaki A. (1971). Arylation of Olefin with Aryl Iodide
Catalyzed by Palladium. BCSJ..

[9] Heck R. F. (1969). Mechanism
of Arylation and Carbomethoxylation of Olefins with Organopalladium
Compounds. J. Am. Chem. Soc..

[10] Heck R. F. (1971). Electronic
and Steric Effects in the Olefin Arylation and Carboalkoxylation Reactions
with Organopalladium Compounds. J. Am. Chem.
Soc..

[11] U.
Luescher M., Gallou F., H. Lipshutz B. (2024). The Impact
of Earth-Abundant Metals as a Replacement for Pd in Cross Coupling
Reactions. Chem. Sci..

[12] Zhang S., He X., Ding Y., Shi Z., Wu B. (2024). Supply and Demand of
Platinum Group Metals and Strategies for Sustainable Management. Renewable and Sustainable Energy Reviews.

[13] Trentin O., Ballesteros-Plata D., Rodríguez-Castellón E., Puppulin L., Selva M., Perosa A., Rodríguez-Padrón D. (2025). Upcycling
of Chitin to Cross-Coupling Catalysts: Tailored Supports and Opportunities
in Mechanochemistry. ChemSusChem.

[14] Nishida E. N., Zaramello L., Campedelli R. R., Keller M. H., Moreira R., S. Souza B. (2025). Sustainable
Heck–Mizoroki and Suzuki–Miyaura
Reactions Mediated by Aqueous Palladium Nanoparticles and Imidazolium–Sulfonate
Additives. New J. Chem..

[15] Kobayashi T., Nakajima L. (2021). Sustainable Development Goals for Advanced Materials
Provided by Industrial Wastes and Biomass Sources. Current Opinion in Green and Sustainable Chemistry.

[16] Anastas P., Nolasco M., Kerton F., Kirchhoff M., Licence P., Pradeep T., Subramaniam B., Moores A. (2021). The Power of the United Nations Sustainable Development
Goals in Sustainable Chemistry and Engineering Research. ACS Sustainable Chem. Eng..

[17] Liu C., Xu W., Xiang D., Luo Q., Zeng S., Zheng L., Tan Y., Ouyang Y., Lin H. (2020). Palladium Immobilized on 2,2′-Dipyridyl-Based
Hypercrosslinked Polymers as a Heterogeneous Catalyst for Suzuki–Miyaura
Reaction and Heck Reaction. Catal. Lett..

[18] Gan W., Xu H., Jin X., Cao X., Gao H. (2020). Recyclable Palladium-Loaded
Hyperbranched Polytriazoles as Efficient Polymer Catalysts for Heck
Reaction. ACS Appl. Polym. Mater..

[19] Nishida E. N., Leopoldino E. C., Zaramello L., Centurion H. A., Gonçalves R. V., Affeldt R. F., Campos C. E. M., Souza B. S. (2022). An Imidazole-Rich
Pd­(II)-Polymer Pre-Catalyst for the Suzuki-Miyaura Coupling: Stability
Influenced by Dissolved Oxygen and Reactants Concentration. ChemCatChem..

[20] Wang G., Chen Z., Yan G., Hu J. (2022). Palladium Nanoparticles
Immobilized on COF-Modified Honeycomb Chitosan Microcapsules as Catalysts
for the Suzuki Reaction and p-Nitrophenol Reduction. New J. Chem..

[21] Xu X.-L., Wang N.-N., Zou Y.-H., Qin X., Wang P., Lu X.-Y., Zhang X.-Y., Sun W.-Y., Lu Y. (2024). N, N’-Bidentate
Ligand Anchored Palladium Catalysts on MOFs for Efficient Heck Reaction. Nat. Commun..

[22] Zheng D.-Y., Bai R., Li M., Wang R., Gu Y. (2023). Dipolar Microenvironment
Enhanced Catalytic Activity of Pd Nanoparticles in MOF Channel. ACS Sustainable Chem. Eng..

[23] Moradi P., Hajjami M., Valizadeh-Kakhki F. (2019). Biochar as
Heterogeneous Support
for Immobilization of Pd as Efficient and Reusable Biocatalyst in
C–C Coupling Reactions. Appl. Organomet.
Chem..

[24] Xie Q., Li J., Wen X., Huang Y., Hu Y., Huang Q., Xu G., Xie Y., Zhou Z. (2022). Carbohydrate-Substituted N-Heterocyclic
Carbenes Palladium Complexes: High Efficiency Catalysts for Aqueous
Suzuki–Miyaura Reaction. Carbohydr. Res..

[25] Schultz D., Campeau L.-C. (2020). Harder, Better,
Faster. Nat.
Chem..

[26] Ashraf M., Ahmad M. S., Inomata Y., Ullah N., Tahir M. N., Kida T. (2023). Transition Metal Nanoparticles as
Nanocatalysts for Suzuki, Heck
and Sonogashira Cross-Coupling Reactions. Coord.
Chem. Rev..

[27] Vásquez-Céspedes S., Betori R. C., Cismesia M. A., Kirsch J. K., Yang Q. (2021). Heterogeneous
Catalysis for Cross-Coupling Reactions: An Underutilized Powerful
and Sustainable Tool in the Fine Chemical Industry?. Org. Process Res. Dev..

[28] Eremin D. B., Ananikov V. P. (2017). Understanding Active
Species in Catalytic Transformations:
From Molecular Catalysis to Nanoparticles, Leaching, “Cocktails”
of Catalysts and Dynamic Systems. Coord. Chem.
Rev..

[29] Sruthi P. R., Malini S., Silpa S., Anas S. (2025). A Novel Reusable Polymer
Supported Palladium Catalyst for Heck Reaction. J. Organomet. Chem..

[30] Yu R., Lan Y., Huang G., Chen C., Ran M., Sun W. (2026). Polydopamine-Mediated
Anchoring of Pd on Magnetic Ni@CNTs: A Robust Core-Shell Nanocatalyst
for Recyclable Heck Cross-Coupling. Colloids
Surf., A.

[31] Lan Y., Ma Y., Hou Q., Luo Z., Wang L., Ran M., Dai T. (2024). Immobilization
of Palladium Nanoparticles on Polydopamine Spheres
with Superior Activity and Reusability in Heck Reaction. J. Catal..

[32] Zhang F., Zhou Y., Wang S., Zhao Y., Wu X., Chen R. (2024). Palladium-Loading Ceramic Catalytic Membrane Reactors
for Mizoroki–Heck
Reaction. Synthesis.

[33] Zhang H., Ye N., Dong R., Li Q., Wang Z., Gao Z., Ji X., Liu L., Ma J., Tong Z. (2024). A Novel Ti^3+^-Mediated Approach for Supporting
Palladium on Anodic TiO2 Nanotubes:
Toward Efficient and Recyclable Catalysis in the Heck Reaction. Applied Catalysis A: General.

[34] Li B., Zeng H. C. (2020). Minimalization of
Metallic Pd Formation in Suzuki Reaction
with a Solid-State Organometallic Catalyst. ACS Appl. Mater. Interfaces.

[35] Widegren J. A., Finke R. G. (2003). A Review of the
Problem of Distinguishing True Homogeneous
Catalysis from Soluble or Other Metal-Particle Heterogeneous Catalysis
under Reducing Conditions. J. Mol. Catal. A:
Chem..

[36] Choi M., Lee D.-H., Na K., Yu B.-W., Ryoo R. (2009). High Catalytic
Activity of Palladium­(II)-Exchanged Mesoporous Sodalite and NaA Zeolite
for Bulky Aryl Coupling Reactions: Reusability under Aerobic Conditions. Angew. Chem., Int. Ed..

[37] Leopoldino E. C., Pinheiro G., Alves R. J., Gerola A., Souza B. S. (2021). Post-Modified
Polymer with Imidazole Groups as an Efficient and Reusable Heterogeneous
Catalyst for Organophosphate Degradation. Materials
Today Communications.

[38] Prat D., Wells A., Hayler J., Sneddon H., McElroy C. R., Abou-Shehada S., Dunn P. J. (2016). CHEM21 Selection
Guide of Classical-
and Less Classical-Solvents. Green Chem..

[39] Byrne F. P., Jin S., Paggiola G., Petchey T. H. M., Clark J. H., Farmer T. J., Hunt A. J., Robert McElroy C., Sherwood J. (2016). Tools and Techniques
for Solvent Selection: Green Solvent Selection Guides. Sustain Chem. Process.

[40] Hessel V., Nghiep Tran N., Razi Asrami M., Don Tran Q., Long N. V. D., Escribà-Gelonch M., Osorio Tejada J., Linke S., Sundmacher K. (2022). Sustainability
of Green Solvents
– Review and Perspective. Green Chem..

[41] Yamada Y. M. A., Yuyama Y., Sato T., Fujikawa S., Uozumi Y. (2014). A Palladium-Nanoparticle
and Silicon-Nanowire-Array Hybrid: A Platform for Catalytic Heterogeneous
Reactions. Angew. Chem., Int. Ed..

[42] Gole B., Sanyal U., Banerjee R., Mukherjee P. S. (2016). High Loading
of Pd Nanoparticles by Interior Functionalization of MOFs for Heterogeneous
Catalysis. Inorg. Chem..

[43] de
Vries J. G. (2006). A Unifying Mechanism for All High-Temperature Heck
Reactions. The Role of Palladium Colloids and Anionic Species. Dalton Trans..

[44] de
Vries A. H. M., Mulders J. M. C. A., Mommers J. H. M., Henderickx H. J. W., de Vries J. G. (2003). Homeopathic Ligand-Free Palladium as a Catalyst in
the Heck Reaction. A Comparison with a Palladacycle. Org. Lett..

[45] de Vries, J. G. Palladium-Catalysed Coupling Reactions. In Organometallics as Catalysts in the Fine Chemical Industry; Beller, M. , Blaser, H.-U. , Eds.; Topics in Organometallic Chemistry Berlin; Springer: Heidelberg; 2012; pp 1–34.

[46] Polynski M.
V., Ananikov V. P. (2019). Modeling
Key Pathways Proposed for the Formation and
Evolution of “Cocktail”-Type Systems in Pd-Catalyzed
Reactions Involving ArX Reagents. ACS Catal..

[47] Deraedt C., Astruc D. (2014). “Homeopathic” Palladium Nanoparticle
Catalysis of Cross Carbon–Carbon Coupling Reactions. Acc. Chem. Res..

[48] Hu Y., Wong M. J., Lipshutz B. H. (2022). Ppm Pd-Containing
Nanoparticles as
Catalysts for Negishi Couplings ··· *in Water*. Angew. Chem. Int. Ed.

[49] Newton O. J., Hellgardt K., Richardson J., Hii K. K. M. (2023). ‘Homeopathic’
Palladium Catalysis? The Observation of Distinct Kinetic Regimes in
a Ligandless Heck Reaction at (Ultra-)­Low Catalyst Loadings. J. Catal..

[50] Weck M., Jones C. W. (2007). Mizoroki–Heck Coupling Using
Immobilized Molecular
Precatalysts: Leaching Active Species from Pd Pincers, Entrapped Pd
Salts, and Pd NHC Complexes. Inorg. Chem..

[51] Gnad C., Abram A., Urstöger A., Weigl F., Schuster M., Köhler K. (2020). Leaching Mechanism
of Different Palladium Surface Species
in Heck Reactions of Aryl Bromides and Chlorides. ACS Catal..

[52] Prima D. O., Vatsadze S. Z. (2025). Cocktail-Type Catalysis: An Emerging
Concept in Metal-Mediated
Transformations. Organometallics.

[53] Chen T., Pang Y., Ali S. H., Chen L., Li Y., Yan X., Wang B. (2024). Efficient Catalysis of Pd (II) Supported by Thiourea
Covalent Organic Framework in Suzuki Coupling Reaction. Molecular Catalysis.

[54] Li W.-J., Chen Y.-X., Kang S.-L., Mo L.-P., Zhang Z.-H. (2023). Design,
Synthesis and Characterization of Palladium-Functionalized Covalent
Organic Framework and Its Application as Heterogeneous Catalysis for
C–H Arylation of Azoles. Chem. A Eur.
J..

[55] Annapurna M., Vishnuvardhan Reddy P., Singh S. P., Kantam M. L. (2013). Heck Cross-Coupling
of Vinyl Heteroaromatic Compounds with Aryl and Heteroaryl Halides
Using Pd­(II) Complex under Phosphine-Free Conditions. Tetrahedron.

[56] Lv L., Li C.-J. (2021). Ruthenium Catalyzed
β-Selective Alkylation of Vinylpyridines
with Aldehydes/Ketones via N2H4Mediated Deoxygenative Couplings. Chem. Sci..

