# Supplementary material for: Sustainable Mizoroki–Heck Cross-Coupling Using a Pd(II)-Polymer as Precatalyst in 1‑Butanol
Source: ACS Omega. 2026 Jun 15;11(25):37369–77. doi: 10.1021/acsomega.6c01810 (PMC13325132; doi:10.1021/acsomega.6c01810)
Supplement: Supplementary file 1 [file ao6c01810_si_001.pdf]

Sustainable Mizoroki–Heck cross-coupling using a Pd(II)-Polymer as precatalyst in 1-butanol

Elvis Naoto Nishida,[a] Laíze Zaramello,[a] Mateus H. Keller,[a] Bruno Luz da Silva,[a] Raphaell Moreira,[b,c] Thorsten M. Gesing,[c,d] Bruno S. Souza[a*]

[a]*Department of Chemistry, Federal University of Santa Catarina, Florianópolis, Santa Catarina, 88040-900, Brazil.*

[b]*Institute of Applied and Physical Chemistry, Universität Bremen, Leobener Str. 6, Bremen D-28359, Germany.*

[c]*Universität Bremen, MAPEX Center for Materials and Processes, Bibliothekstr. 1, Bremen D-28359, Germany.*

[d]*Universität Bremen, Institute of Inorganic Chemistry and Crystallography, Leobener Str. 7, Bremen D-28359, Germany.*

*bruno.souza@ufsc.br

Electronic Supplementary Information (ESI)

Figure S1. TEM micrographs from the particles collected after the first (A) and (B) fourth cycle in H_2O-*i*PrOH in MH reaction between iodobenzene and ethyl acrylate. Histograms obtained from the approximate counting of 250 spherical particles. **p.S2**

Figure S2. Comparison of FTIR spectra from fresh Pd/PECIm, PEMA hydrolyzed and Pd/PECIm after fourth cycle. **p.S2**

Figure S3. XPS spectra from (A) fresh Pd/PECIm and (B) after one cycle of MH reaction between iodobenzene and ethyl acrylate in 1-ButOH **p.S3**

Figure S4. ^{1}H NMR (400 MHz, $CDCl_3$) of methyl cinnamate **p.S4**

Figure S5. ^{13}C NMR (100 MHz, $CDCl_3$) of methyl cinnamate **p.S5**

Figure S6. ^{1}H NMR (400 MHz, $CDCl_3$) of ethyl cinnamate **p.S6**

Figure S7. ^{13}C NMR (100 MHz, $CDCl_3$) of ethyl cinnamate **p.S7**

Figure S8. ^{1}H NMR (400 MHz, $CDCl_3$) of *tert*-butyl cinnamate **p.S8**

Figure S9. ^{13}C NMR (100 MHz, $CDCl_3$) of *tert*-butyl cinnamate **p.S9**

Figure S10. ^{1}H NMR (400 MHz, $CDCl_3$) of *trans*-stilbene **p.S10**

Figure S11. ^{13}C NMR (100 MHz, $CDCl_3$) of *trans*-stilbene **p.S11**

Figure S12. ^{1}H NMR (400 MHz, $CDCl_3$) of *trans*-4-acetylstilbene **p.S12**

Figure S13. ^{13}C NMR (100 MHz, $CDCl_3$) of *trans*-4-acetylstilbene **p.S13**

Figure S14. ^{1}H NMR (400 MHz, $CDCl_3$) of *trans*-4-methoxystilbene **p.S14**

Figure S15. ^{13}C NMR (100 MHz, $CDCl_3$) of *trans*-4-methoxystilbene **p.S15**

Figure S16. ^{1}H NMR (400 MHz, $CDCl_3$) of ethyl 4-methoxycinnamate **p.S16**

Figure S17. ^{13}C NMR (100 MHz, $CDCl_3$) of ethyl 4-methoxycinnamate **p.S17**

Figure S18. ^{1}H NMR (400 MHz, $CDCl_3$) of ethyl 4-nitrocinnamate **p.S18**

Figure S19. ^{13}C NMR (100 MHz, $CDCl_3$) of ethyl 4-nitrocinnamate **p.S19**

Figure S20. ^{1}H NMR (400 MHz, $CDCl_3$) of ethyl 2-methylcinnamate **p.S20**

Figure S21. ^{13}C NMR (100 MHz, $CDCl_3$) of ethyl 2-methylcinnamate **p.S21**

Figure S22. ^{1}H NMR (400 MHz, $CDCl_3$) of *tert*-butyl 4-methoxycinnamate **p.S22**

Figure S23. ^{13}C NMR (100 MHz, $CDCl_3$) of *tert*-butyl 4-methoxycinnamate **p.S23**

Figure S24. ^{1}H NMR (400 MHz, $CDCl_3$) of *tert*-butyl 2-methylcinnamate **p.S24**

Figure S25. ^{13}C NMR (100 MHz, $CDCl_3$) of *tert*-butyl 2-methylcinnamate **p.S25**

Figure S26. ^{1}H NMR (400 MHz, $CDCl_3$) of *trans*-4-styrylpyridine **p.S26**

Figure S27. ^{13}C NMR (100 MHz, $CDCl_3$) of *trans*-4-styrylpyridine **p.S27**

Figure S28. UV-Vis spectra and calibration curve from ethyl cinnamate in ethanol **p.S28**

Powder X-ray diffraction (XRPD) data refinement **p.S29**

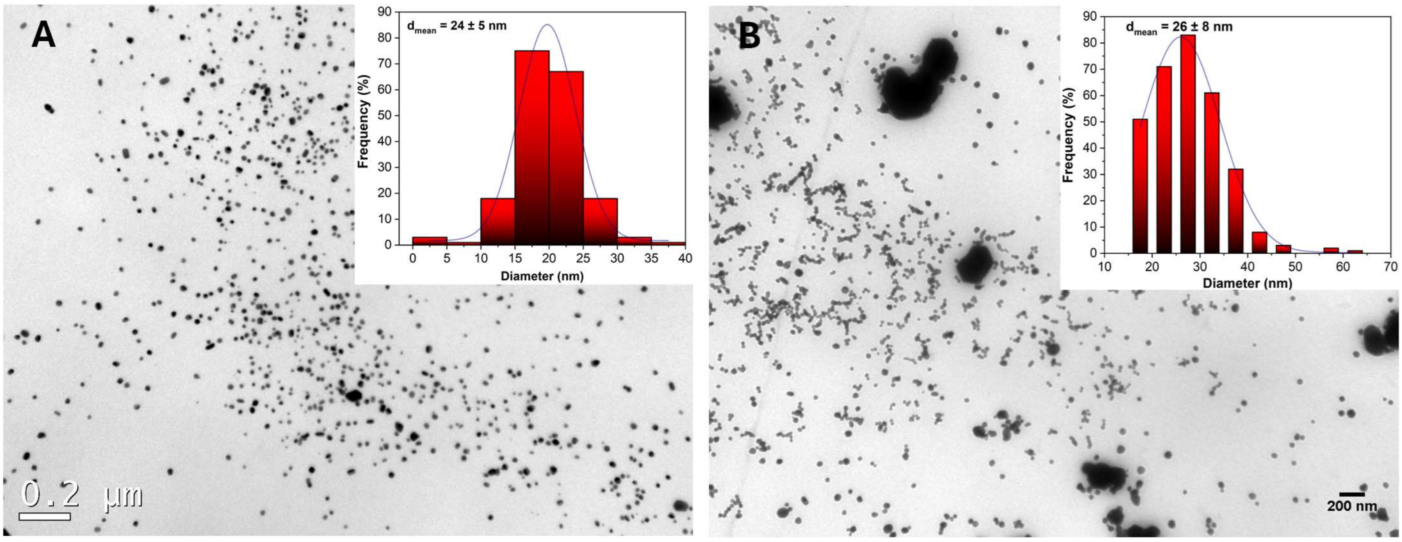

Figure S1. TEM micrographs from the particles collected after the first (A) and (B) fourth cycle in H_2O-*i*PrOH in MH reaction between iodobenzene and ethyl acrylate. Histograms obtained from the approximate counting of 250 spherical particles.

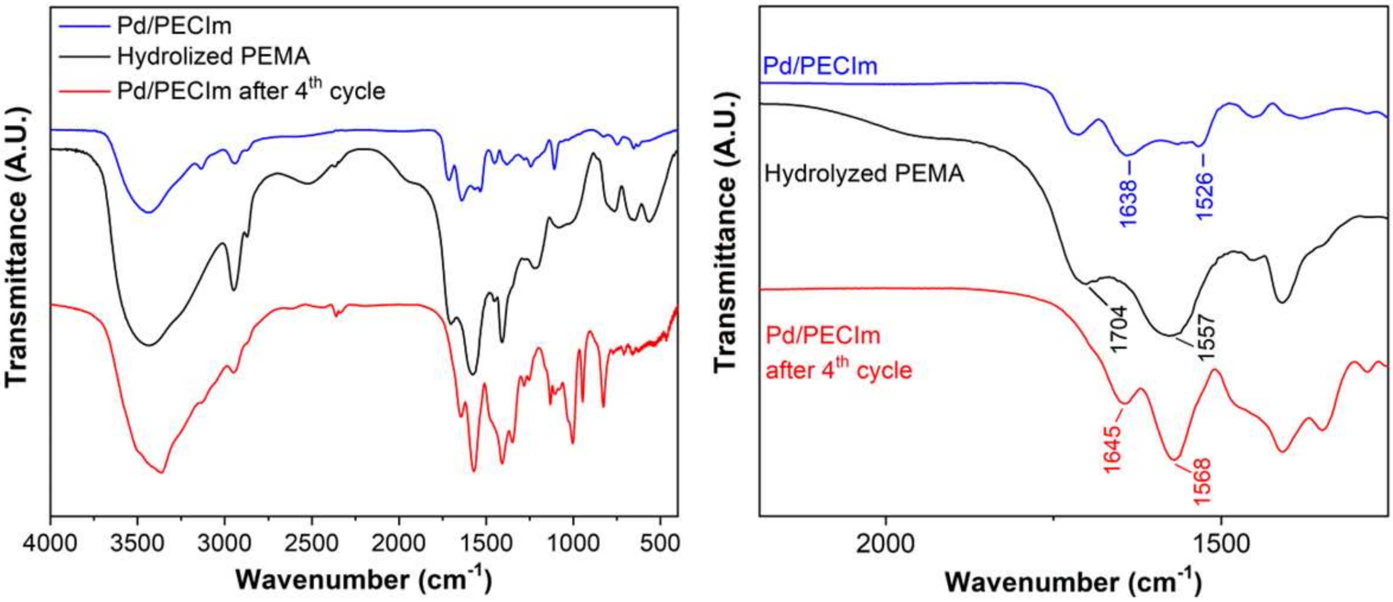

Figure S2. Comparison of FTIR spectra from fresh Pd/PECIm, hydrolized PEMA and Pd/PECIm after fourth cycle. Amplified range from 2000–1500 cm^{-1} corresponding from carbonyl from amide and carboxylate stretching.

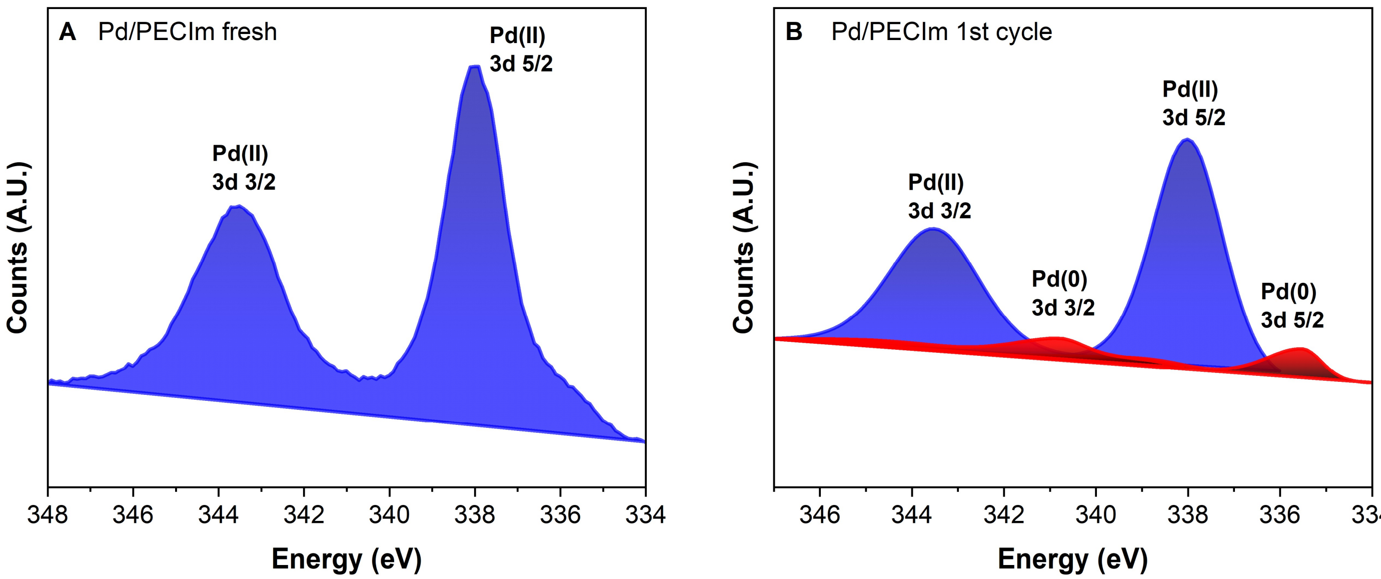

Figure S3. XPS spectra from (A) fresh Pd/PECIm and (B) after one cycle of MH reaction between iodobenzene and ethyl acrylate in 1-ButOH.

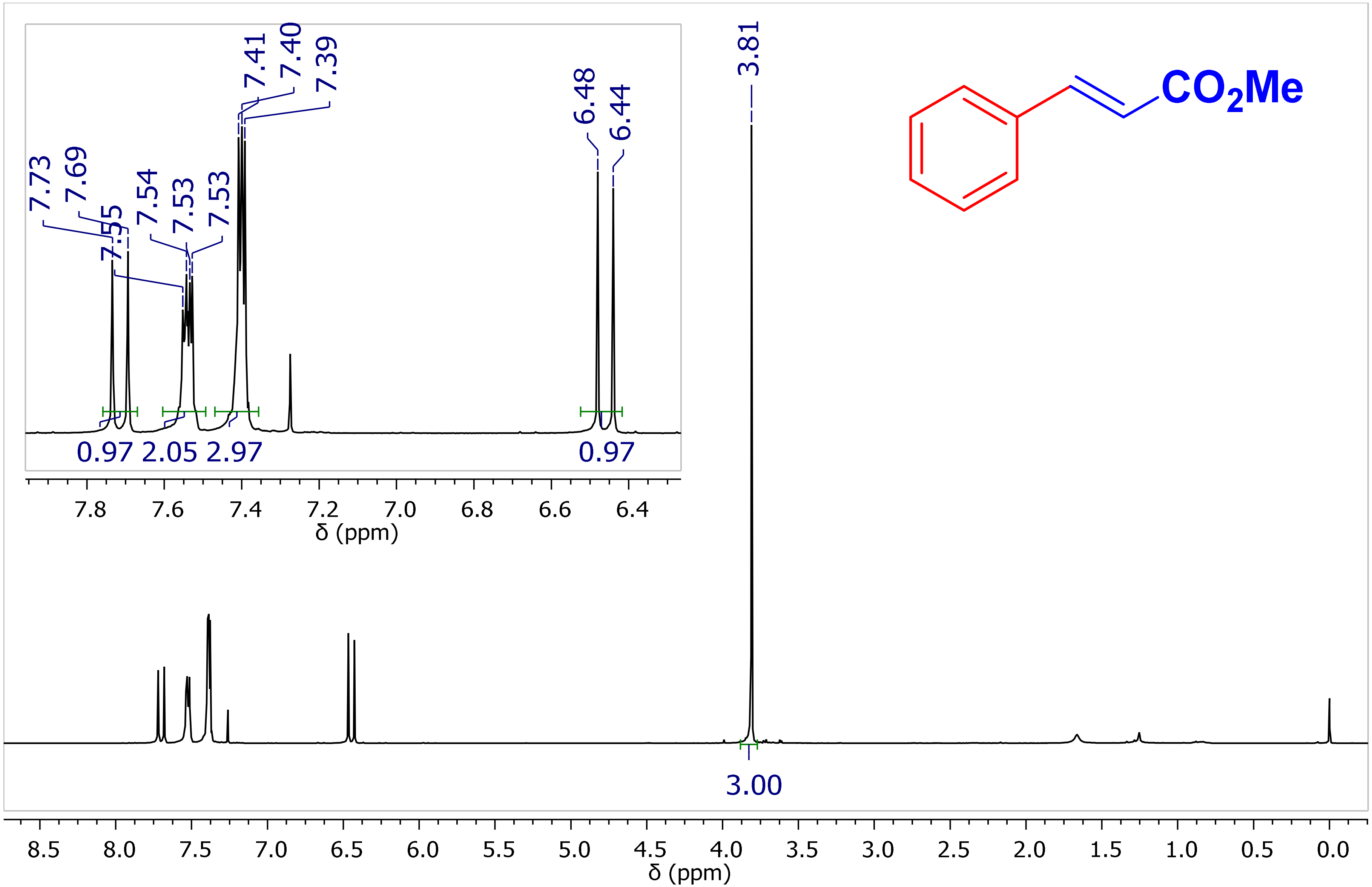

Figure S4. ^{1}H NMR (400 MHz, $CDCl_3$) of methyl cinnamate. δ (ppm) 7.73, 7.69, 7.55, 7.54, 7.53, 7.53, 7.41, 7.40, 7.39, 6.48, 6.44, 3.81. The spectrum is consistent with previously reported data.[1]

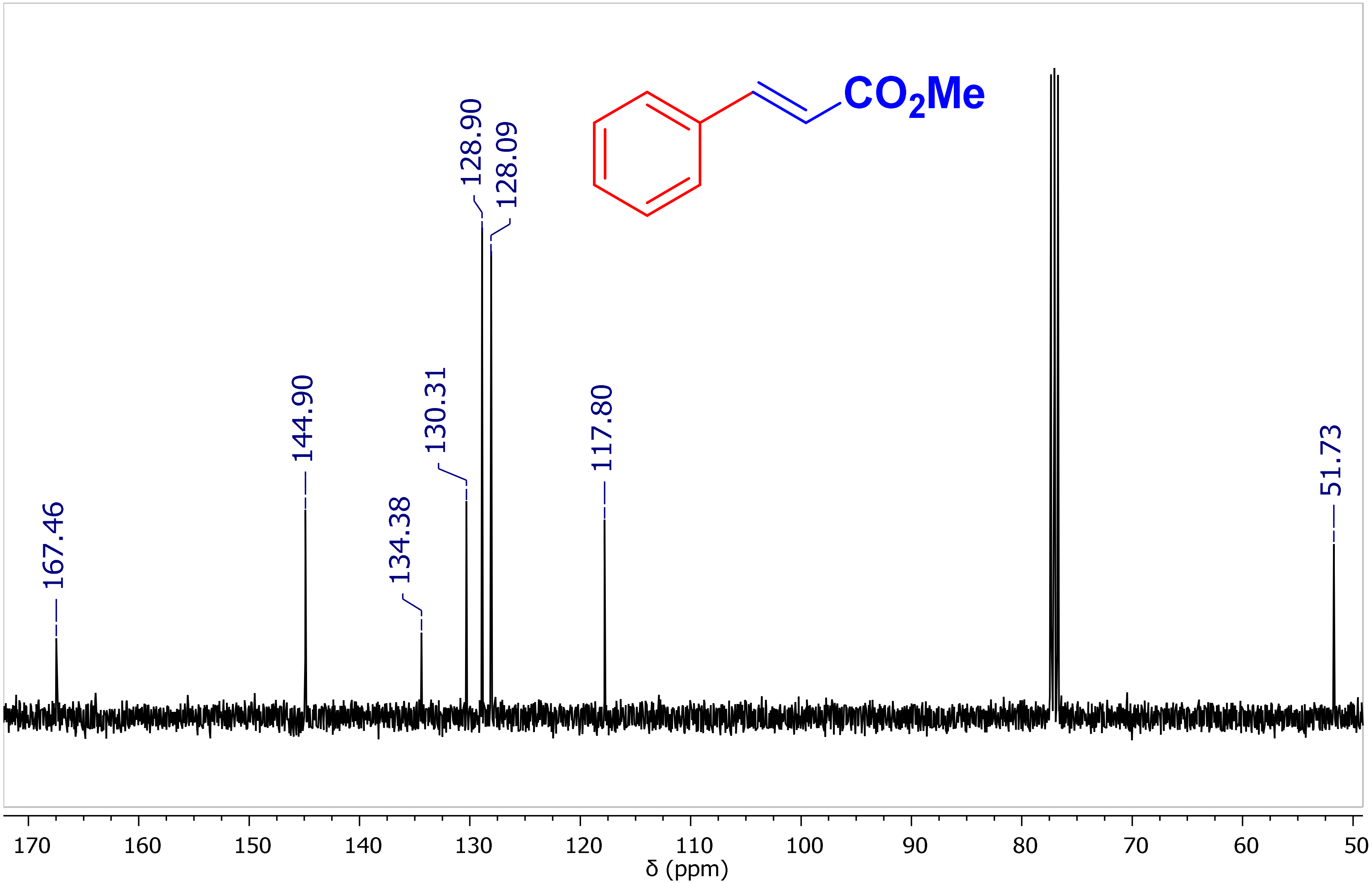

Figure S5. ^{13}C NMR (100 MHz, $CDCl_3$) of methyl cinnamate. δ (ppm) 167.46, 144.90, 134.38, 130.31, 128.90, 128.09, 117.80, 51.73. The spectrum is consistent with previously reported data.[1]

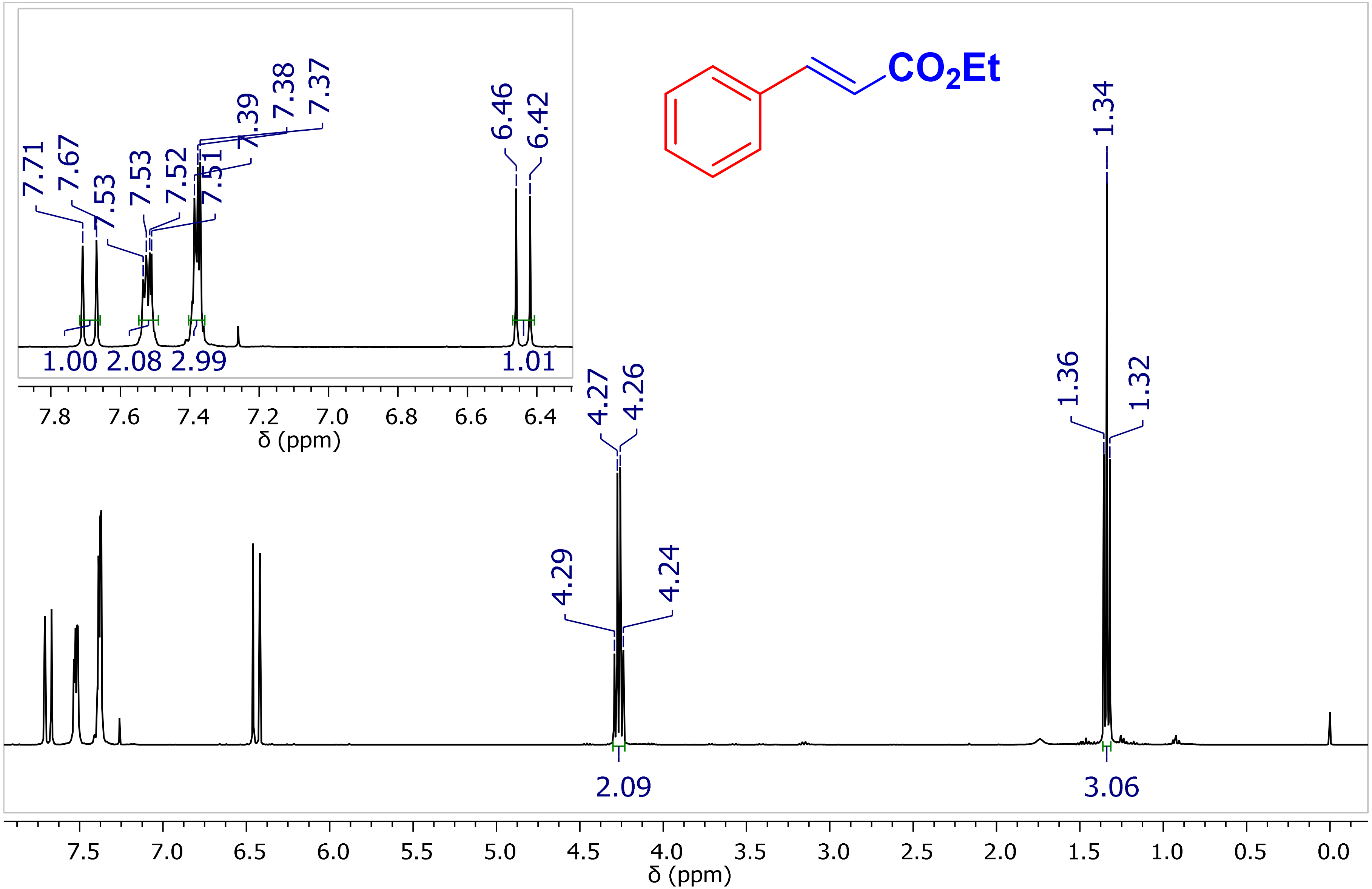

Figure S6. ^{1}H NMR (400 MHz, $CDCl_3$) of ethyl cinnamate. δ (ppm) 7.71, 7.67, 7.53, 7.53, 7.52, 7.51, 7.39, 7.38, 7.37, 6.46, 6.42, 4.29, 4.27, 4.26, 4.24, 1.36, 1.34, 1.32. The spectrum is consistent with previously reported data.[2]

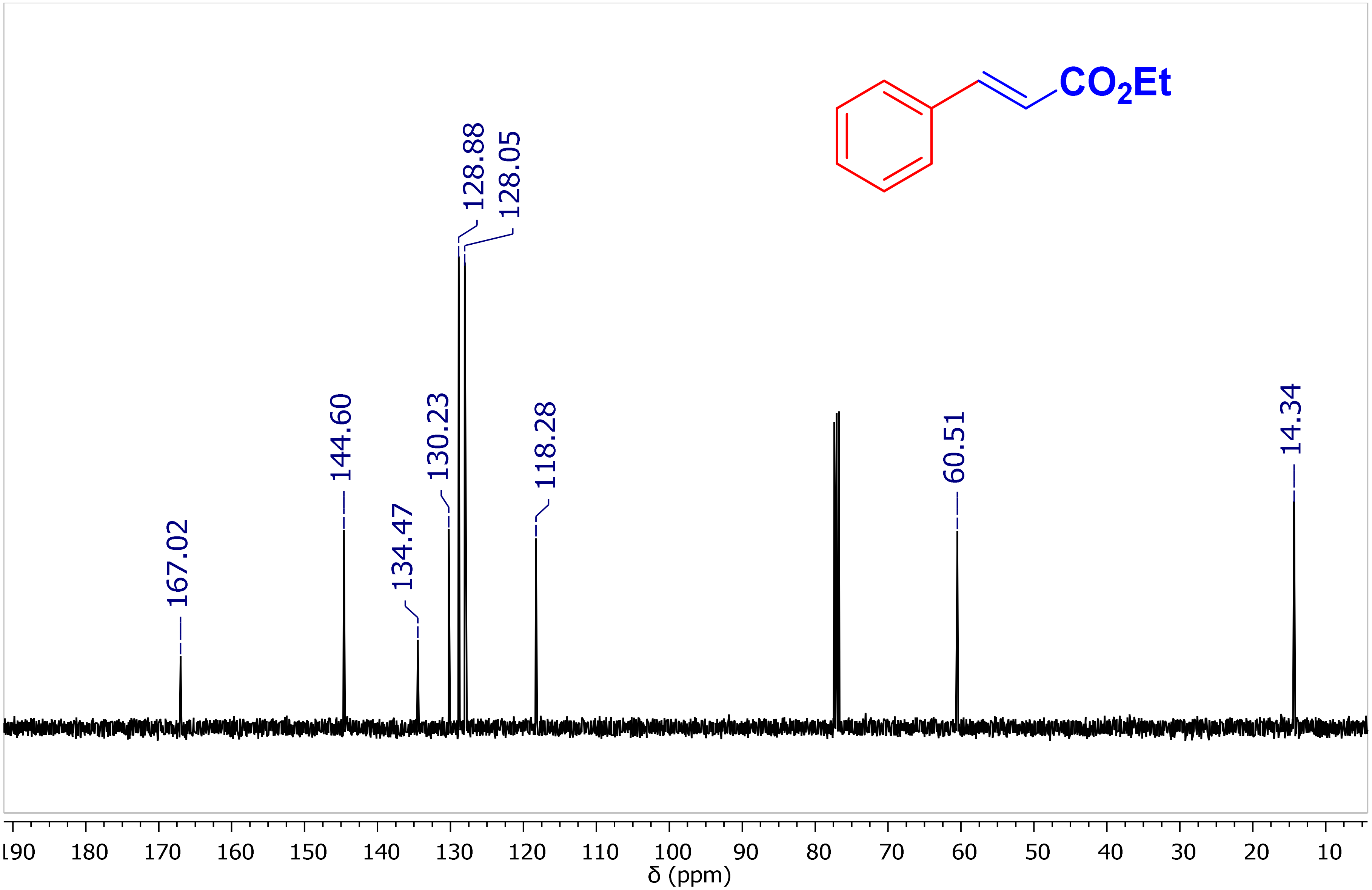

Figure S7. ^{13}C NMR (100 MHz, $CDCl_3$) of ethyl cinnamate. δ (ppm) 167.02, 144.60, 134.47, 130.23, 128.88, 128.05, 118.28, 60.51, 14.34. The spectrum is consistent with previously reported data.[2]

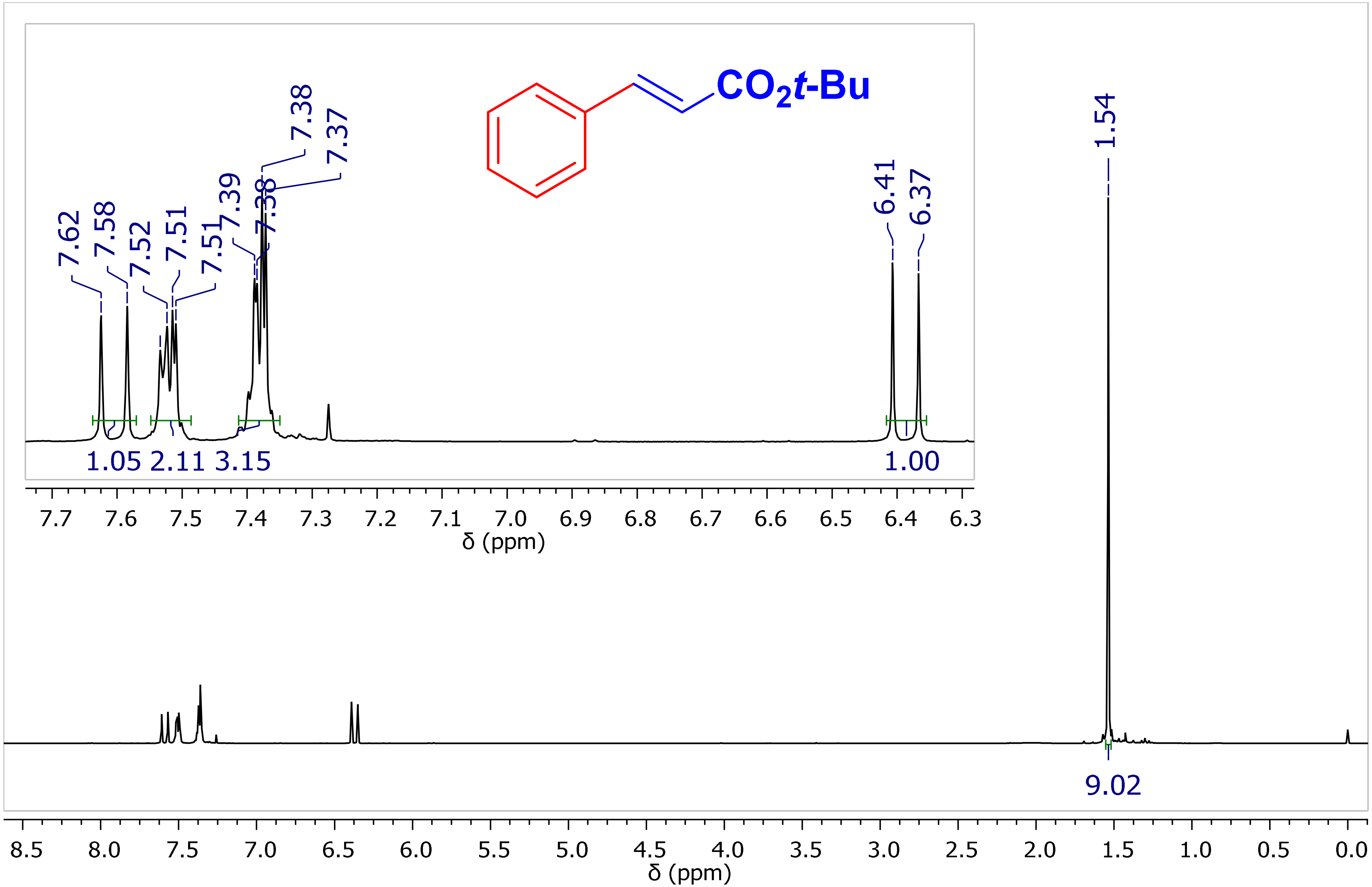

Figure S8. ^{1}H NMR (400 MHz, $CDCl_3$) of *tert*-butyl cinnamate. δ (ppm) 7.62, 7.58, 7.52, 7.51, 7.51, 7.39, 7.38, 7.38, 7.37, 6.41, 6.37, 1.54. The spectrum is consistent with previously reported data.[3]

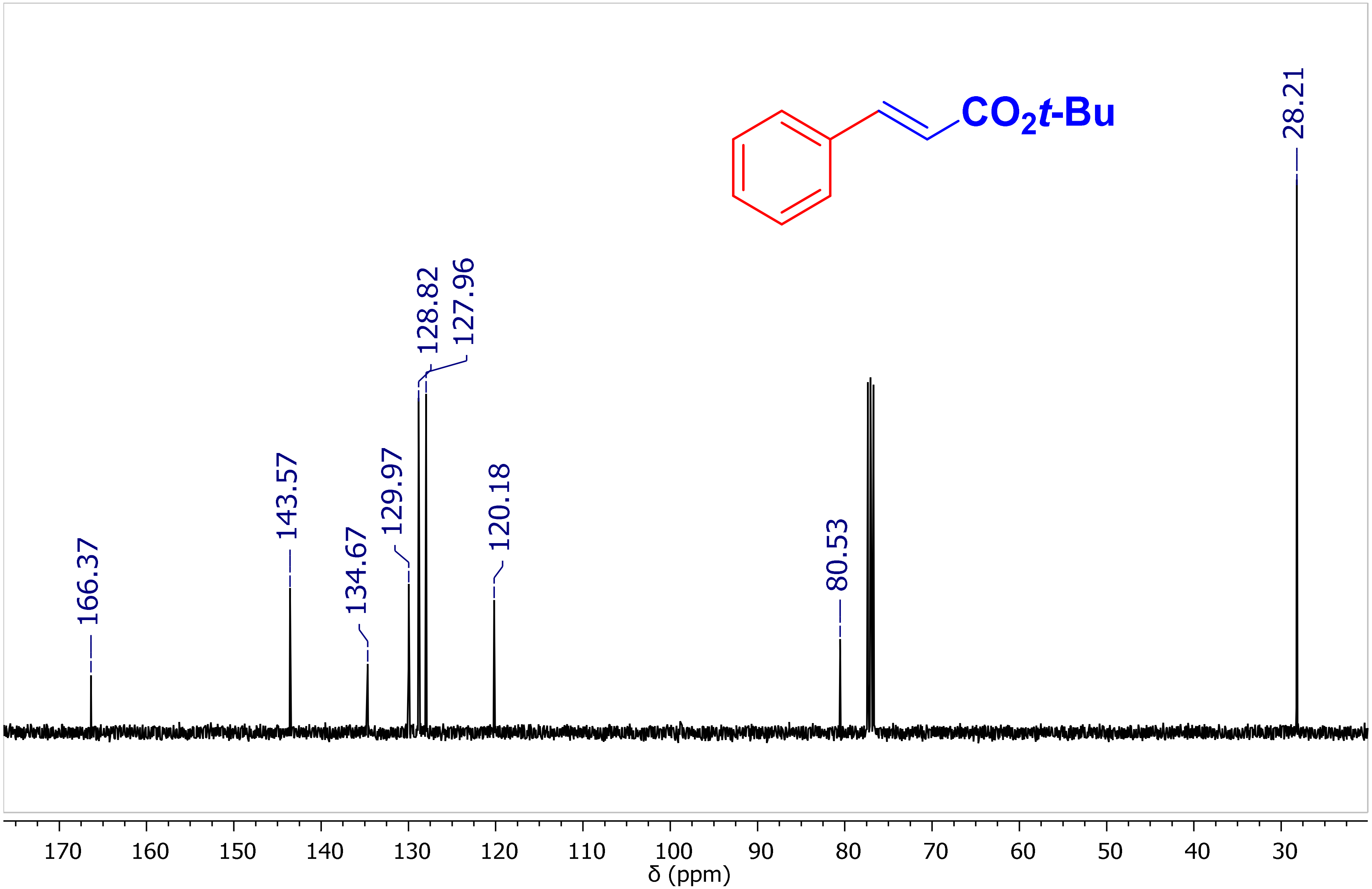

Figure S9. ^{13}C NMR (100 MHz, $CDCl_3$) of *tert*-butyl cinnamate. δ (ppm) 166.37, 143.57, 134.67, 129.97, 128.82, 127.96, 120.18, 80.53, 28.21. The spectrum is consistent with previously reported data.[3]

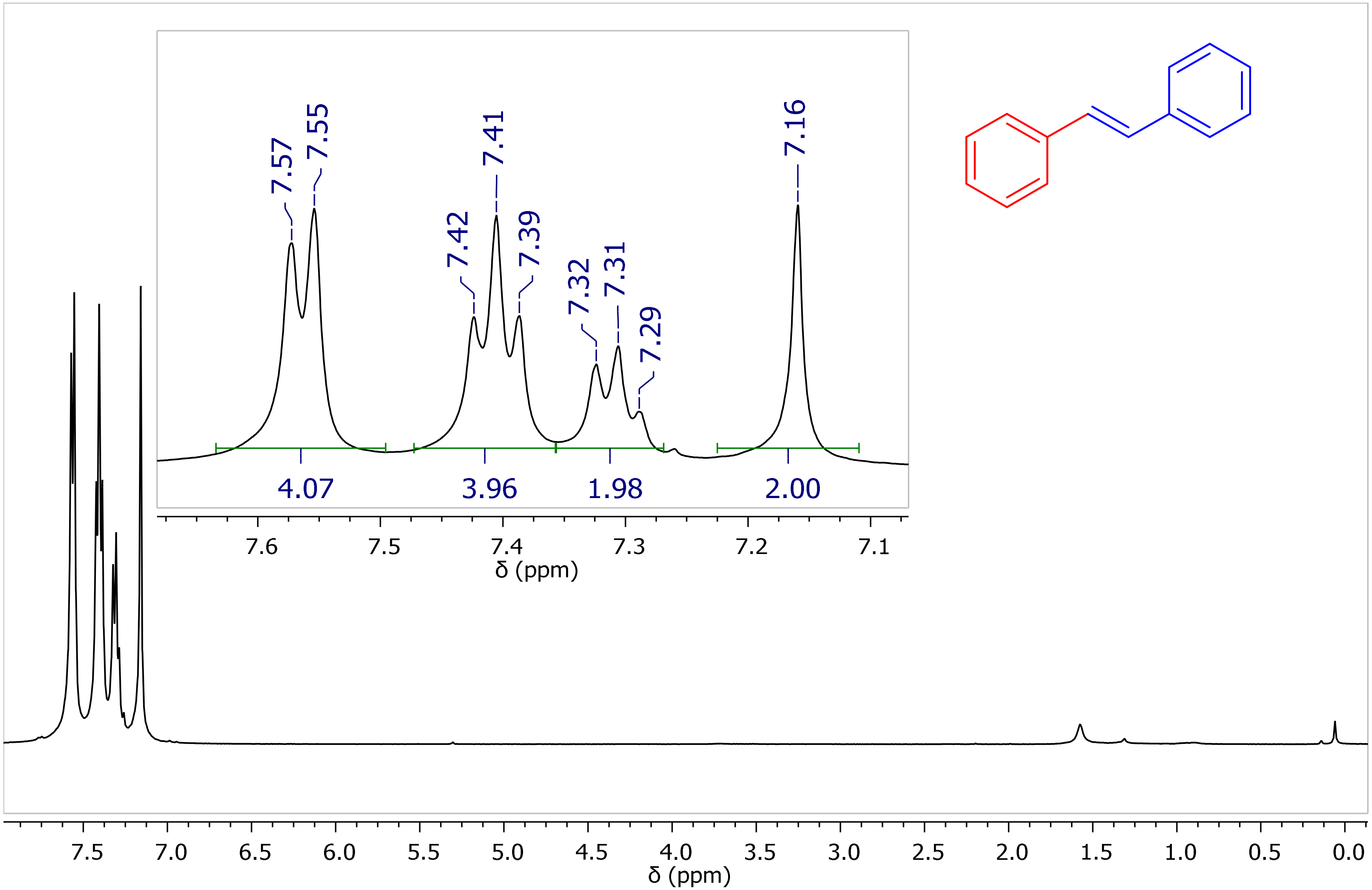

Figure S10. ^{1}H NMR (400 MHz, $CDCl_3$) of *trans*-stilbene. δ (ppm) 7.57, 7.55, 7.42, 7.41, 7.39, 7.32, 7.31, 7.29, 7.16. The spectrum is consistent with previously reported data.[1]

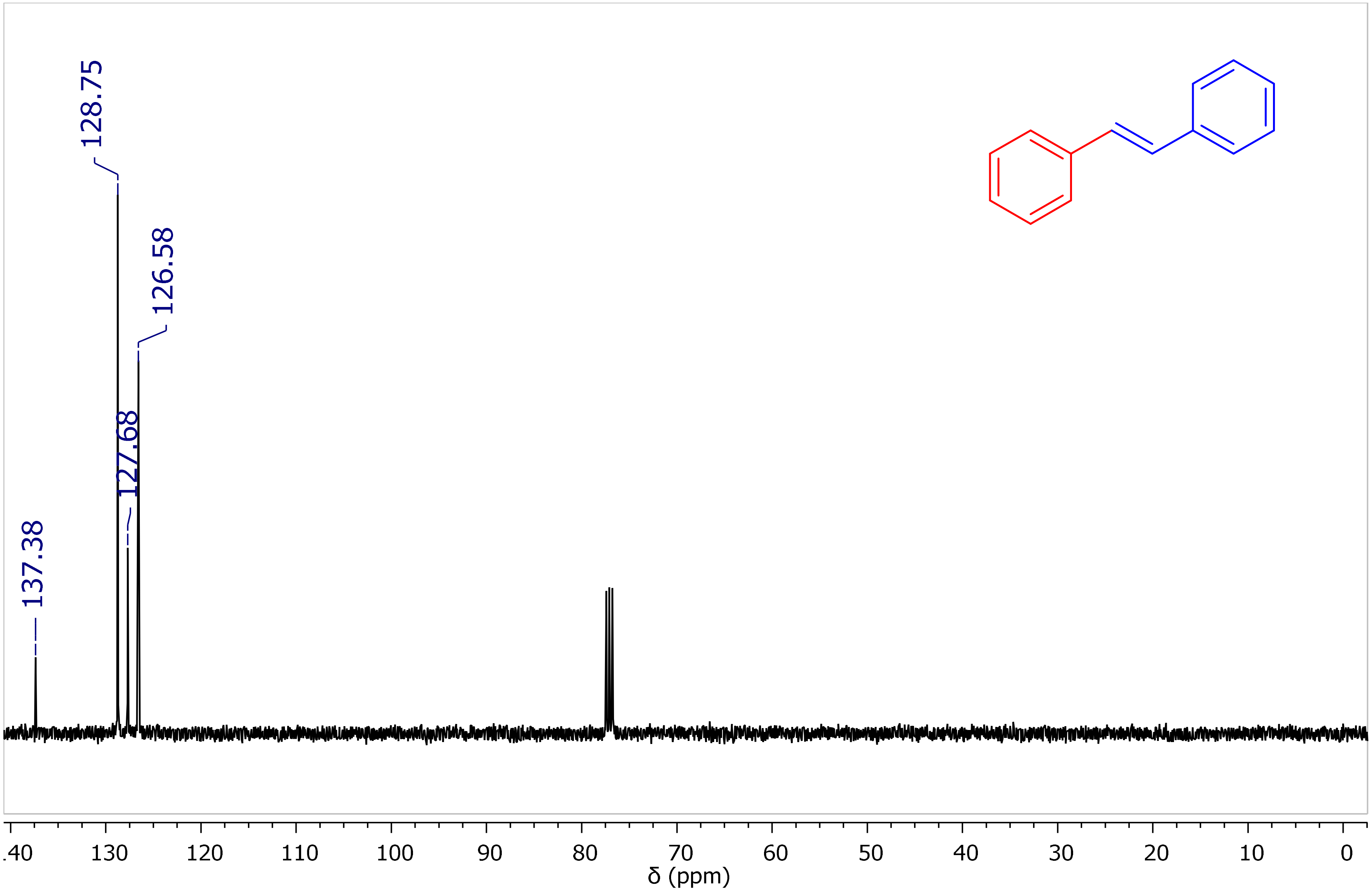

Figure S11. ^{13}C NMR (100 MHz, $CDCl_3$) of *trans*-stilbene. δ (ppm) 137.38, 128.75, 127.68, 126.58. The spectrum is consistent with previously reported data.[1]

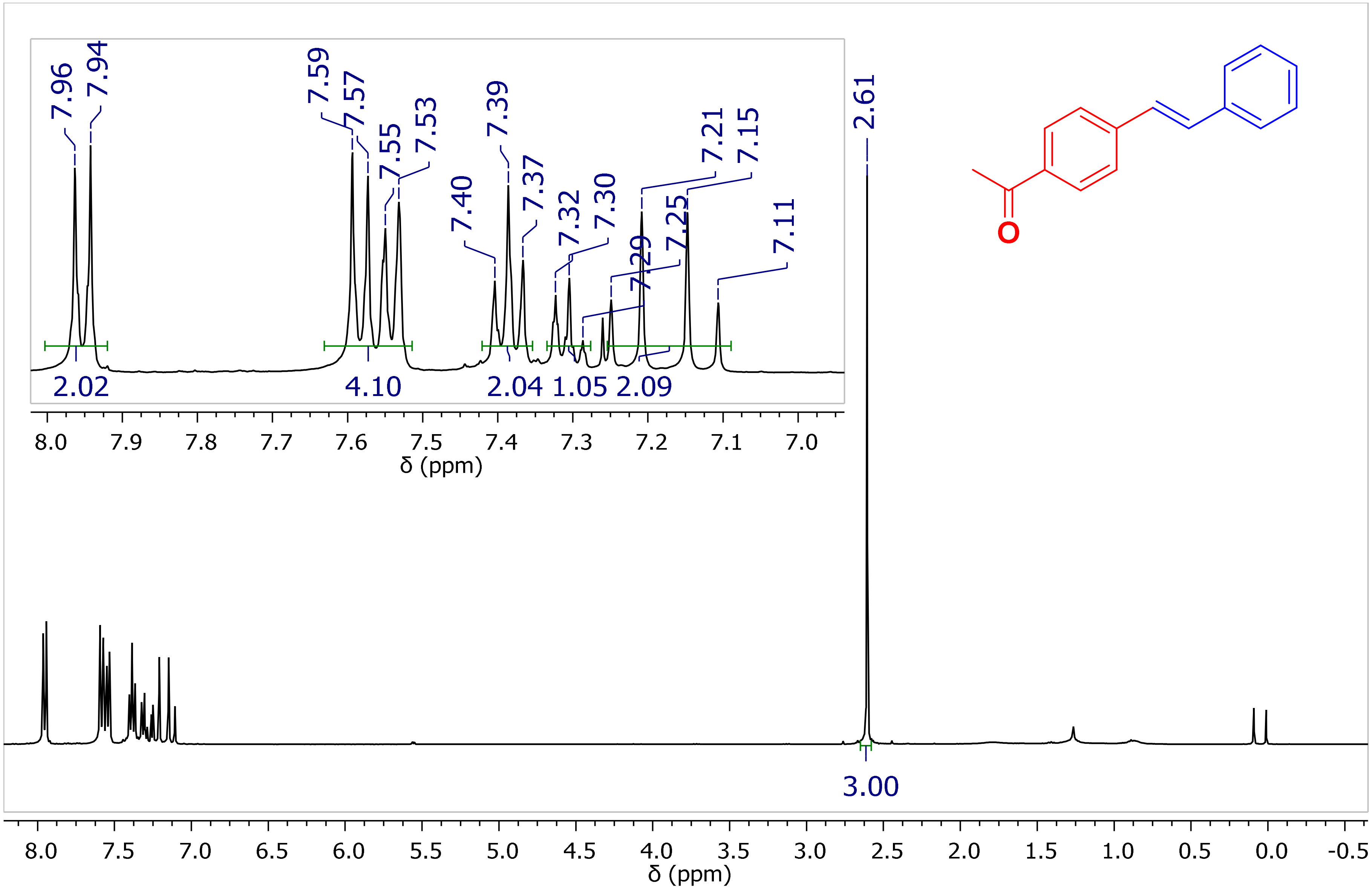

Figure S12. ^{1}H NMR (400 MHz, CDCl3) of *trans*-4-acetylstilbene. δ (ppm). 7.96, 7.94, 7.59, 7.57, 7.55, 7.53, 7.40, 7.39, 7.37, 7.32, 7.30, 7.29, 7.25, 7.21, 7.15, 7.11, 2.61. The spectrum is consistent with previously reported data.[1]

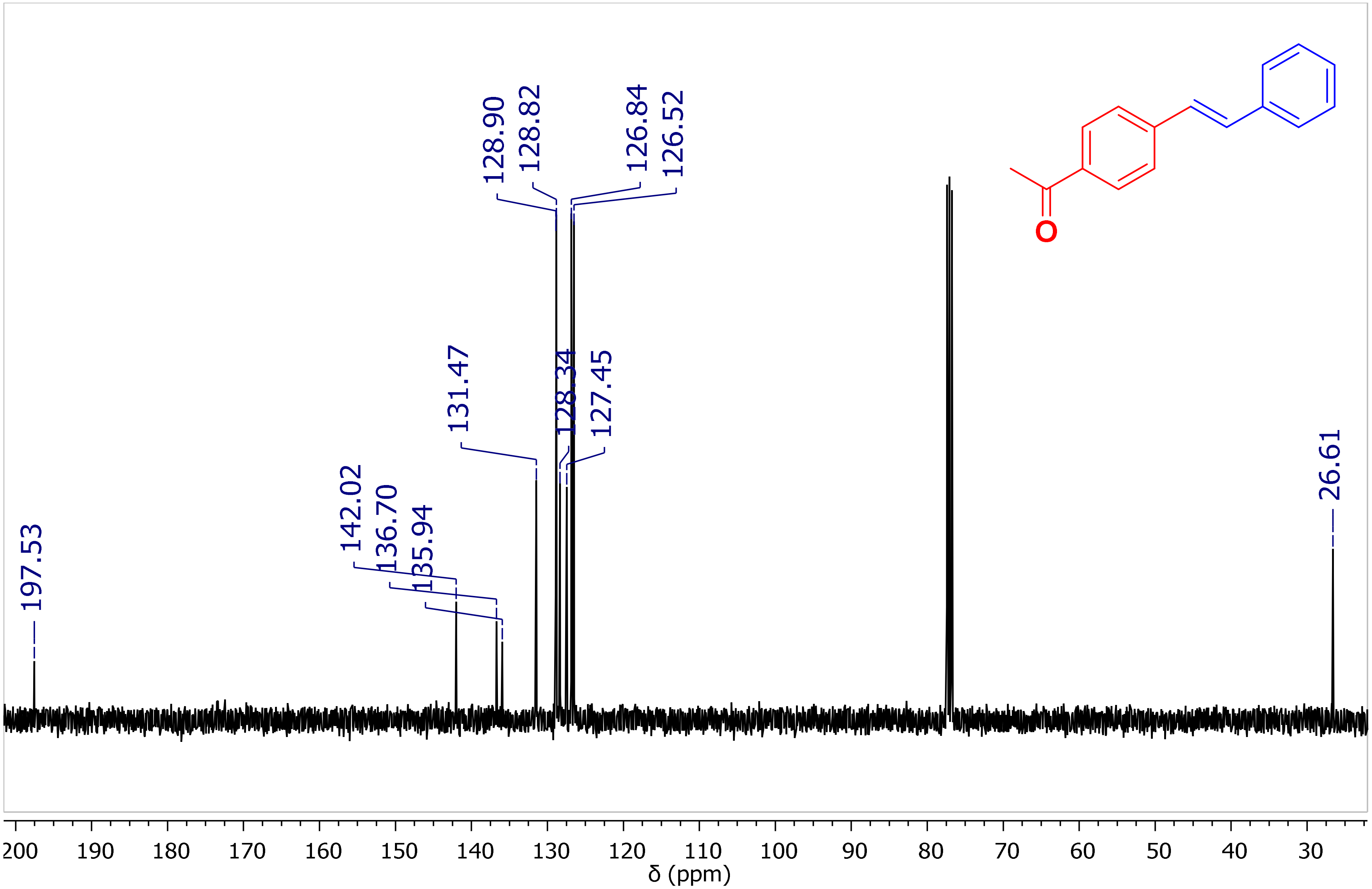

Figure S13. ^{13}C NMR (100 MHz, $CDCl_3$) of *trans*-4-acetylstilbene. δ (ppm) 197.53, 142.02, 136.70, 135.94, 131.47, 128.90, 128.82, 128.34, 127.45, 126.84, 126.52, 26.61. The spectrum is consistent with previously reported data.[1]

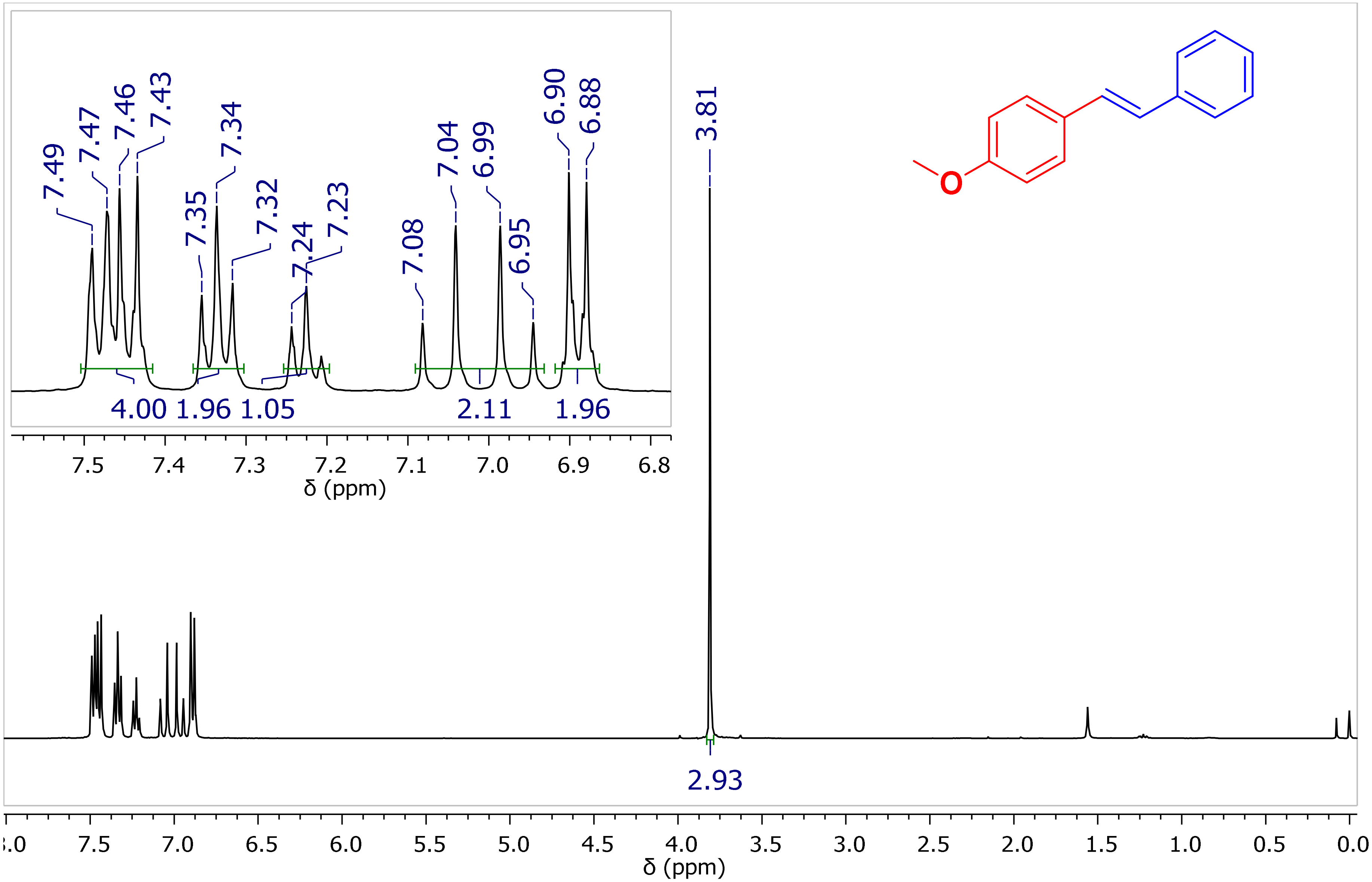

Figure S14. ^{1}H NMR (200 MHz, $CDCl_3$) of *trans*-4-methoxystilbene. δ (ppm) 7.49, 7.47, 7.46, 7.43, 7.35, 7.34, 7.32, 7.24, 7.23, 7.21, 7.08, 7.04, 6.99, 6.95, 6.90, 6.88, 3.81. The spectrum is consistent with previously reported data.[1]

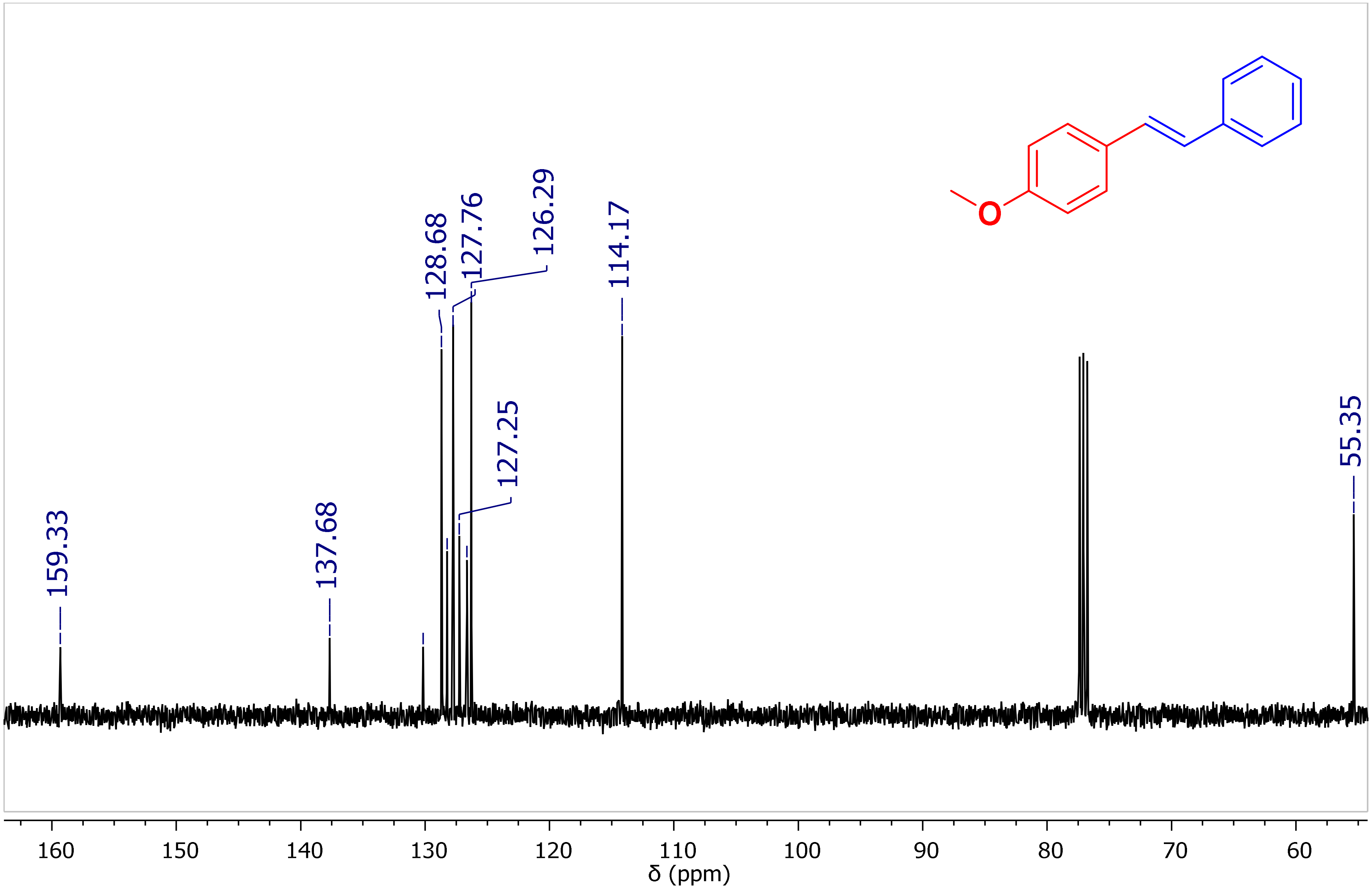

Figure S15. ^{13}C NMR (100 MHz, $CDCl_3$) of *trans*-4-methoxystilbene. δ (ppm) 159.33, 137.68, 130.17, 128.68, 128.24, 127.76, 127.25, 126.64, 126.29, 114.17, 55.35. The spectrum is consistent with previously reported data.[1]

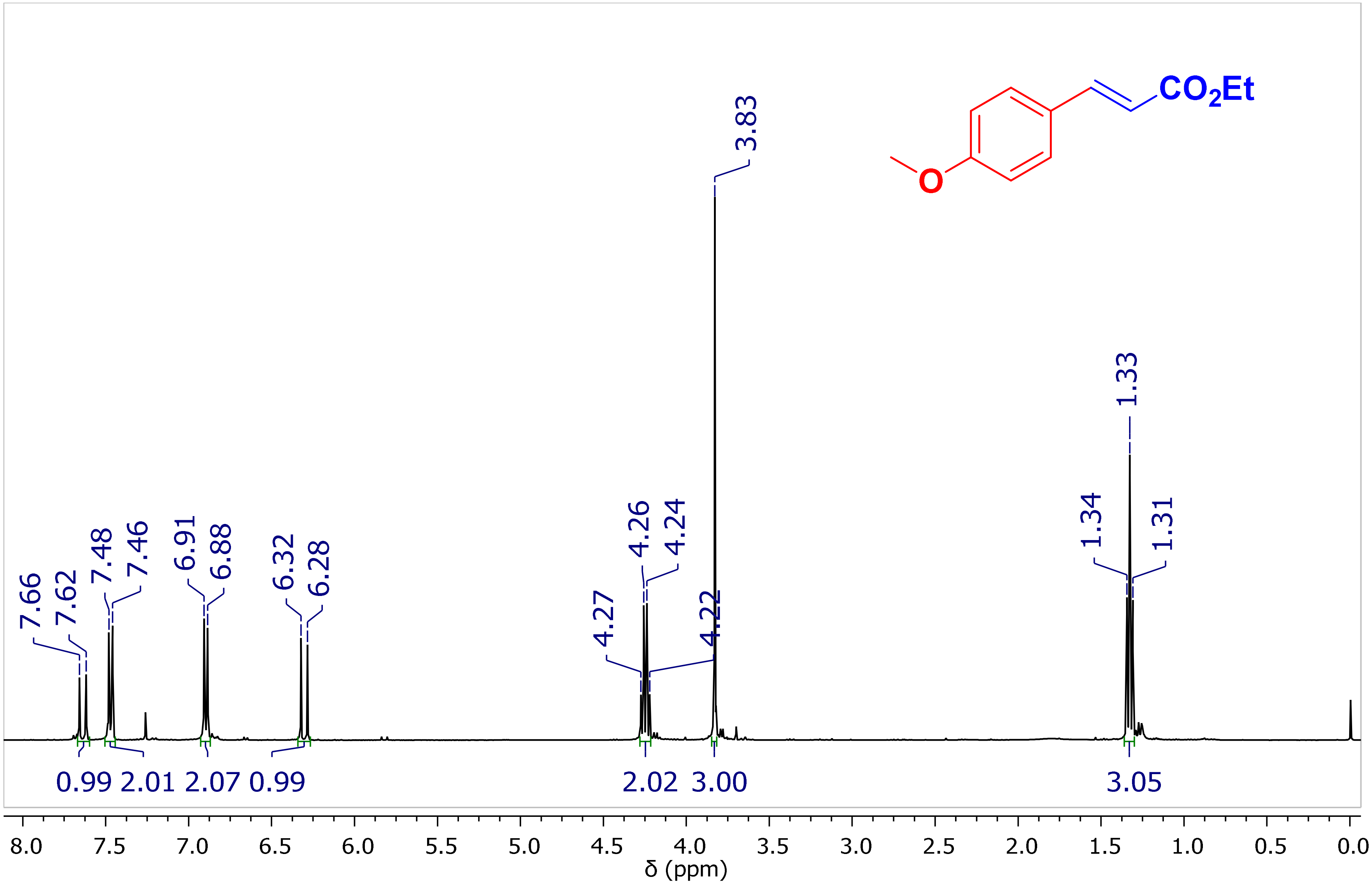

Figure S16. ^{1}H NMR (400 MHz, $CDCl_3$) of ethyl 4-methoxycinnamate. δ (ppm) 7.66, 7.62, 7.48, 7.46, 6.91, 6.88, 6.32, 6.28, 4.27, 4.26, 4.24, 4.22, 3.83, 1.34, 1.33, 1.31. The spectrum is consistent with previously reported data.[1]

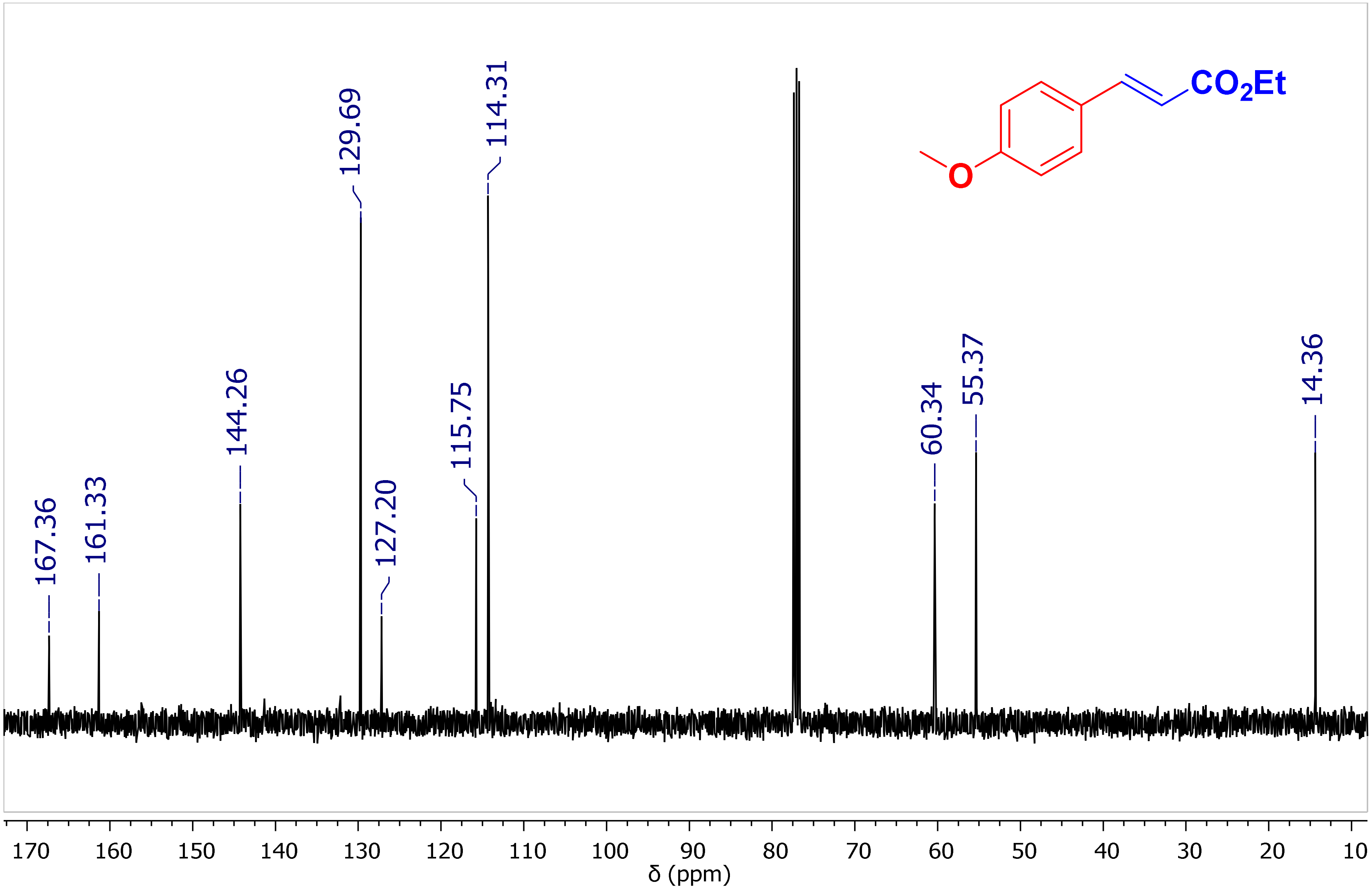

Figure S17. ^{13}C NMR (100 MHz, $CDCl_3$) of ethyl 4-methoxycinnamate. δ (ppm) 167.36, 161.33, 144.26, 129.69, 127.20, 115.75, 114.31, 60.34, 55.37, 14.36. The spectrum is consistent with previously reported data.[1]

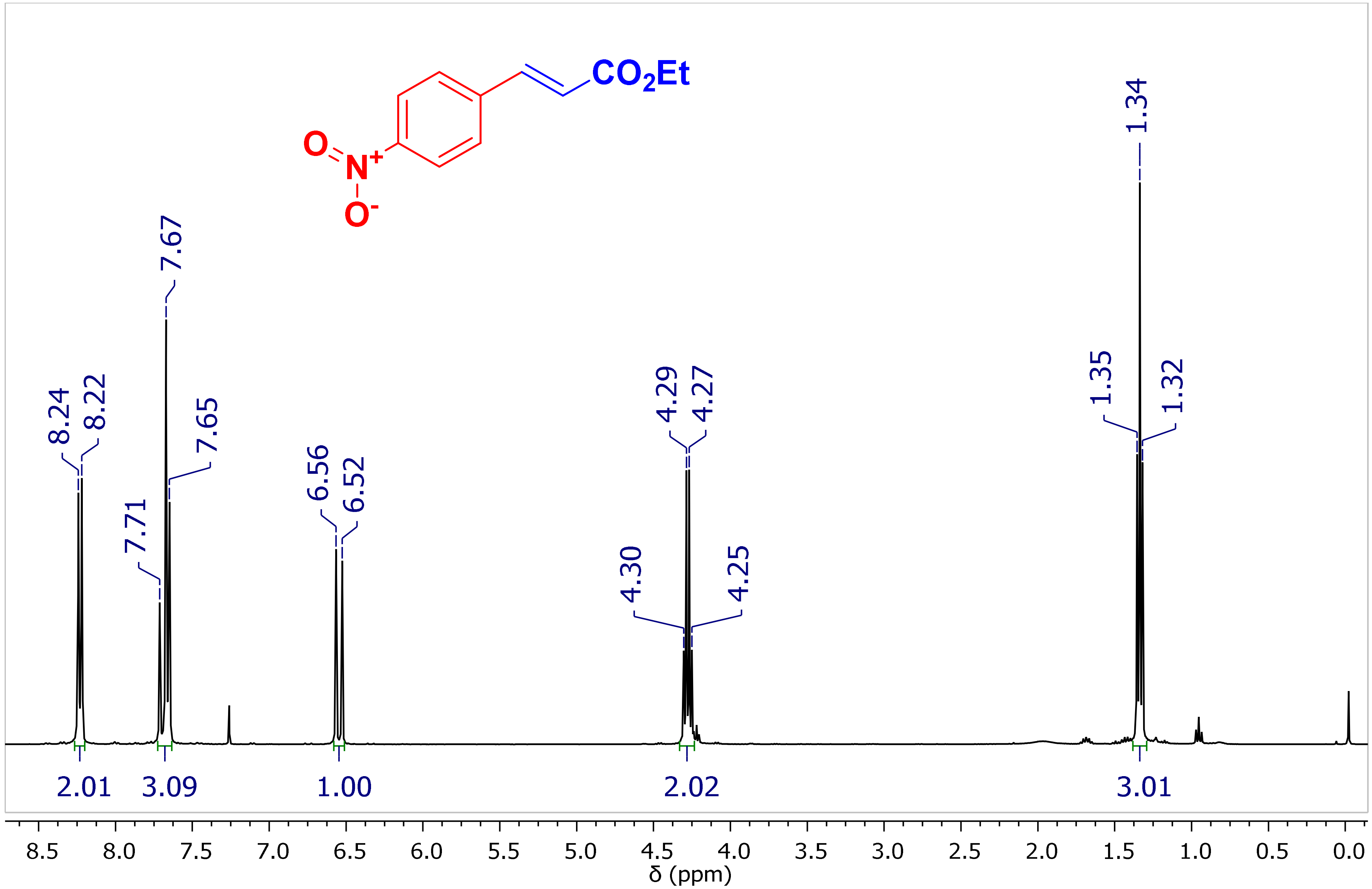

Figure S18. ^{1}H NMR (400 MHz, $CDCl_3$) of ethyl 4-nitrocinnamate. δ (ppm) 8.24, 8.22, 7.71, 7.67, 7.65, 6.56, 6.52, 4.30, 4.29, 4.27, 4.25, 1.35, 1.34, 1.32. The spectrum is consistent with previously reported data.[1]

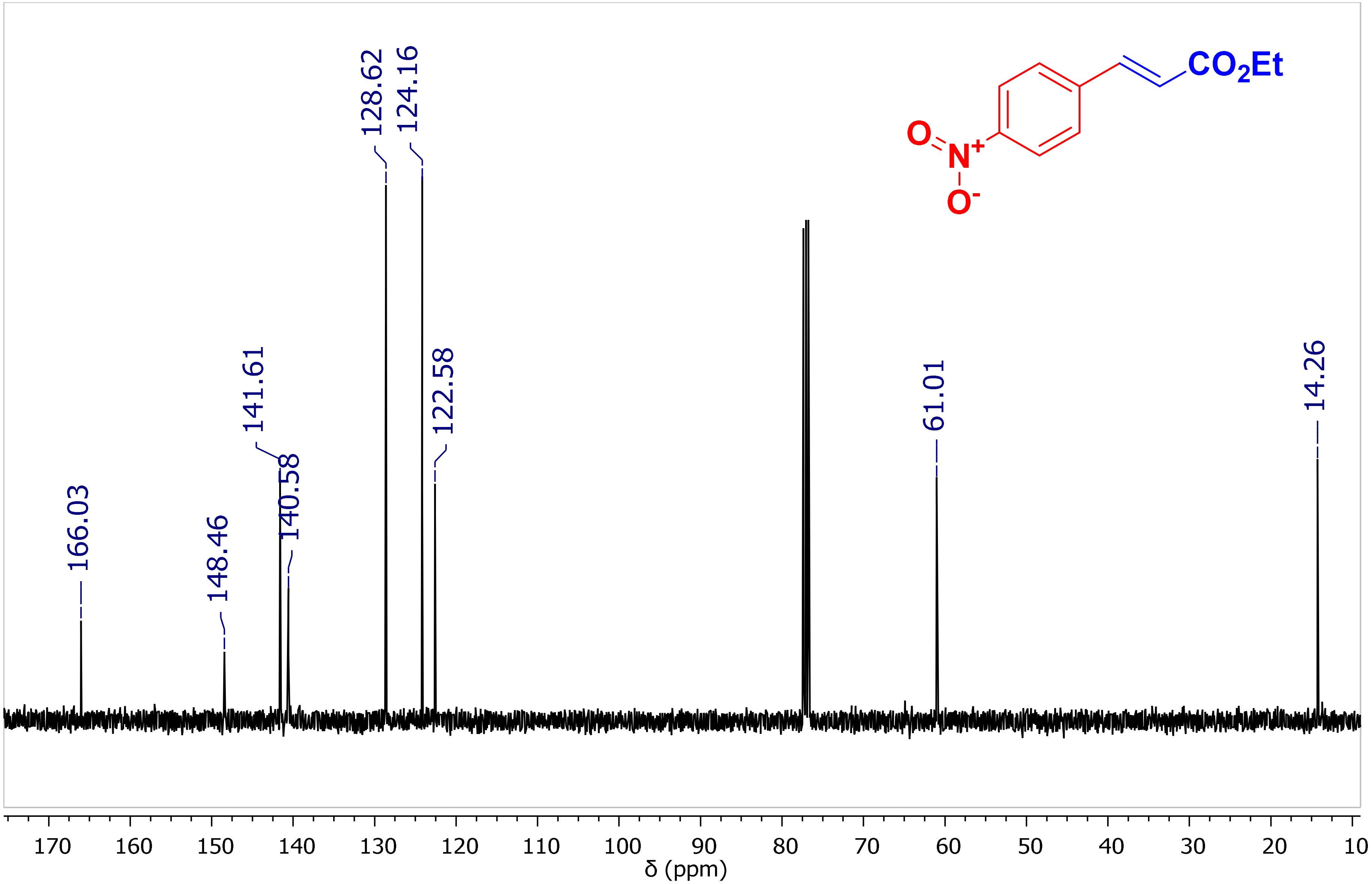

Figure S19. ^{13}C NMR (100 MHz, $CDCl_3$) of ethyl 4-nitrocinnamate. δ (ppm) 166.03, 148.46, 141.61, 140.58, 128.62, 124.16, 122.58, 61.01, 14.26. The spectrum is consistent with previously reported data.[1]

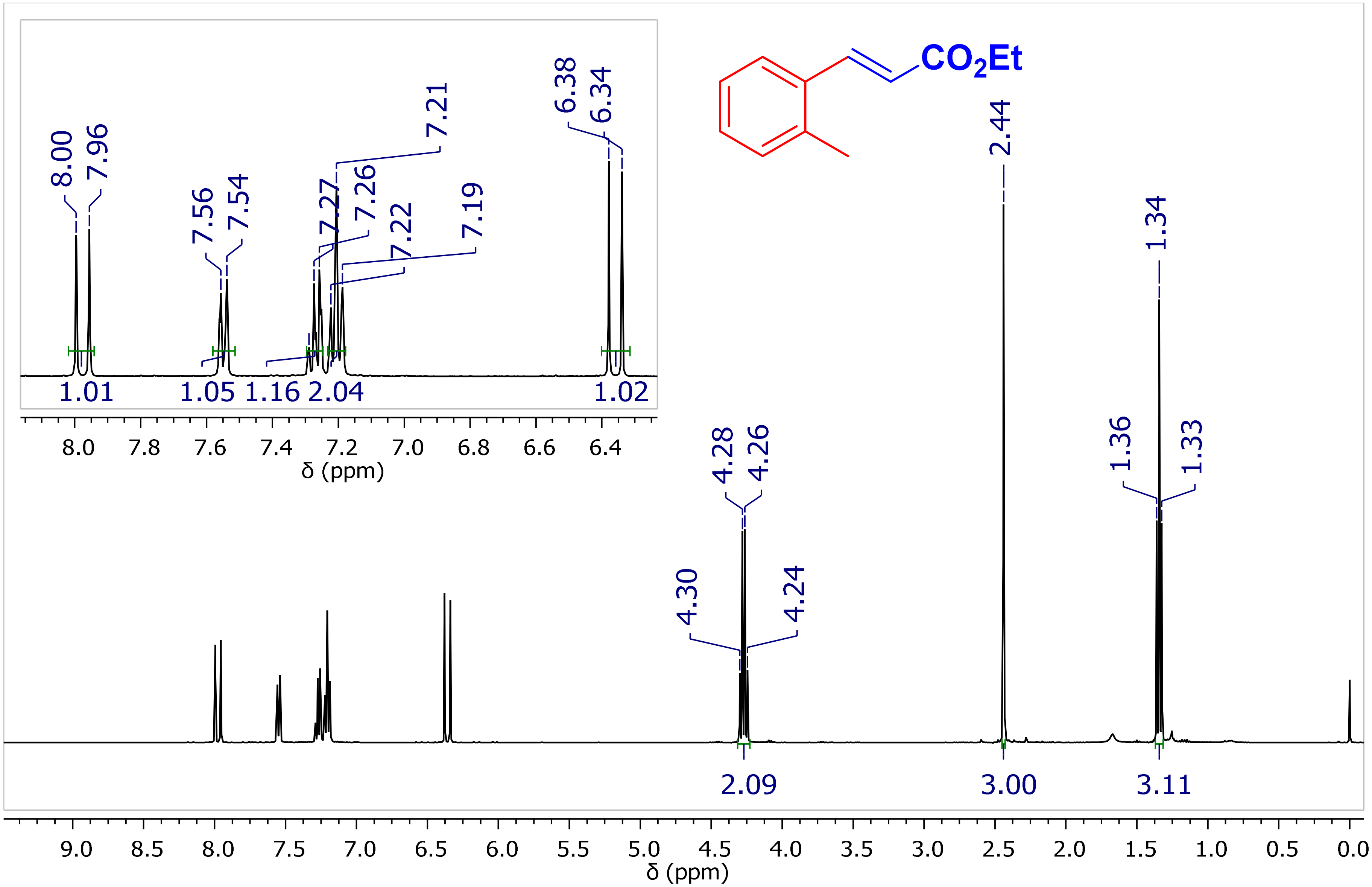

Figure S20. ^{1}H NMR (400 MHz, $CDCl_3$) of ethyl 2-methylcinnamate. δ (ppm) 8.00, 7.96, 7.56, 7.55, 7.27, 7.26, 7.22, 7.21, 7.19, 6.38, 6.34, 4.30, 4.28, 4.26, 4.24, 2.44, 1.36, 1.34, 1.33. The spectrum is consistent with previously reported data.[4]

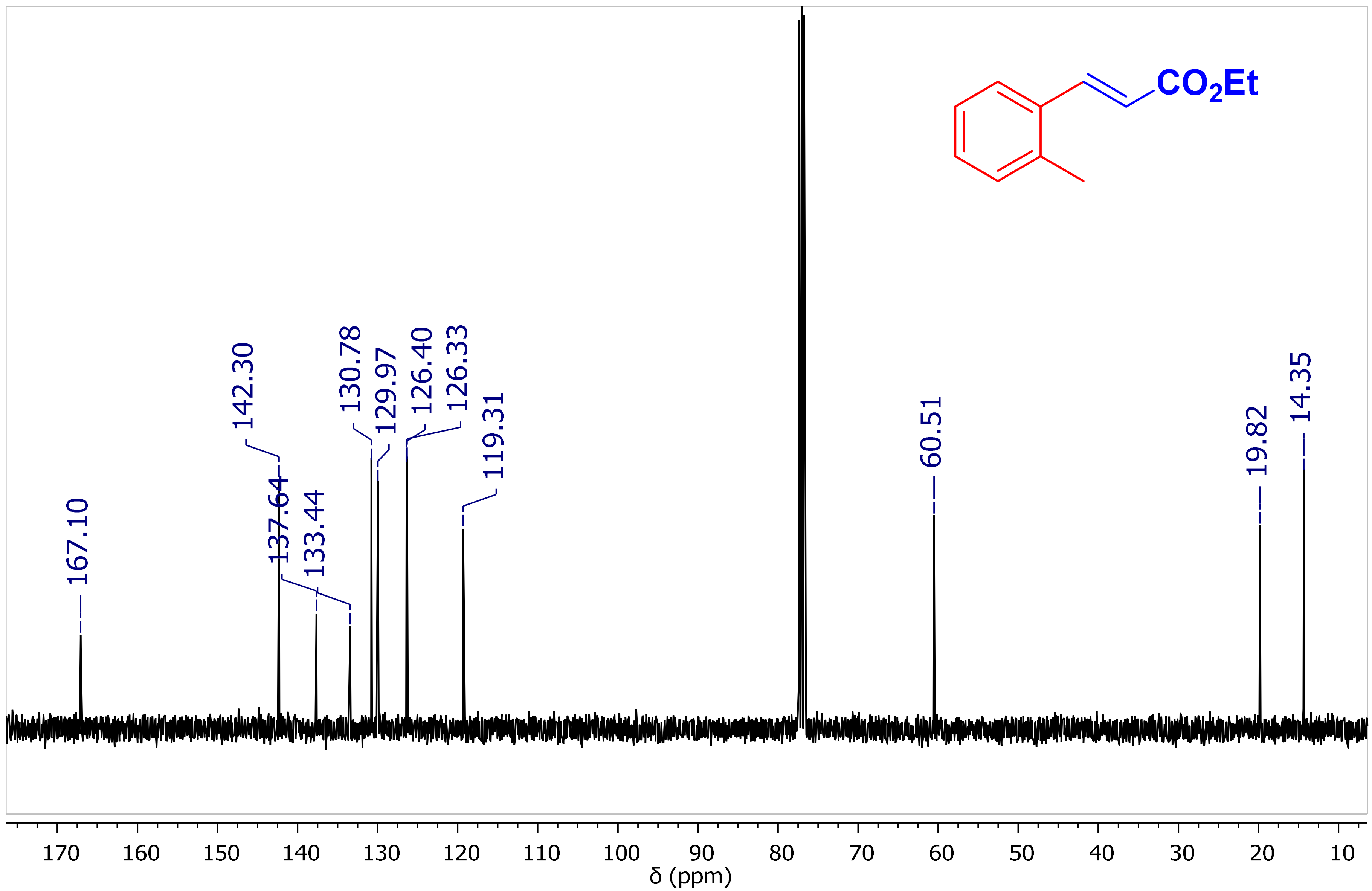

Figure S21. ^{13}C NMR (100 MHz, $CDCl_3$) of ethyl 2-methylcinnamate. δ (ppm) 167.10, 142.30, 137.64, 133.44, 130.78, 129.97, 126.40, 126.33, 119.31, 60.51, 19.82, 14.35. The spectrum is consistent with previously reported data.[4]

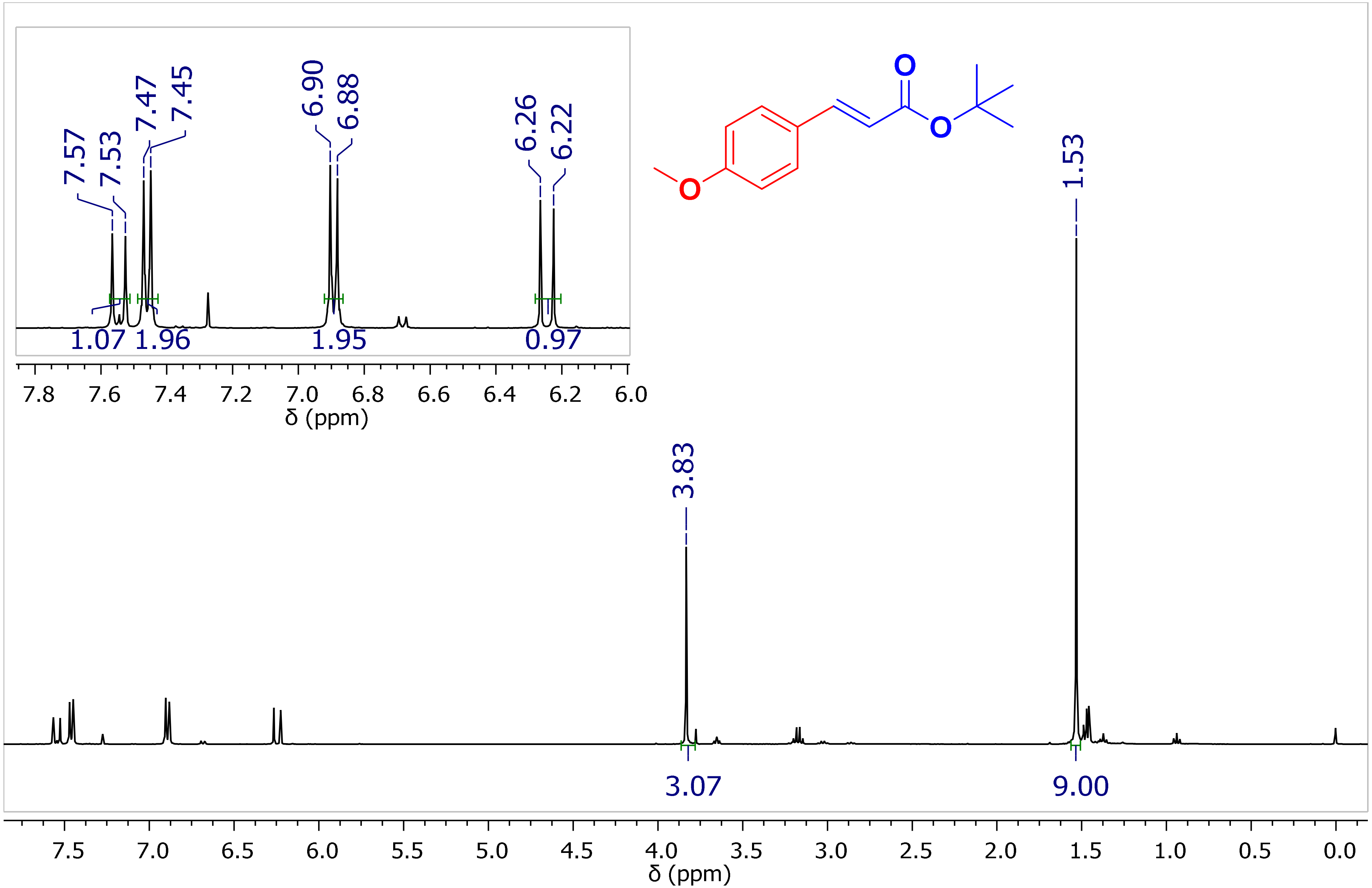

Figure S22. ^{1}H NMR (400 MHz, $CDCl_3$) of *tert*-butyl 4-methoxycinnamate. δ (ppm) 7.57, 7.53, 7.47, 7.45, 6.90, 6.88, 6.26, 6.22, 3.83, 1.53. The spectrum is consistent with previously reported data.[3]

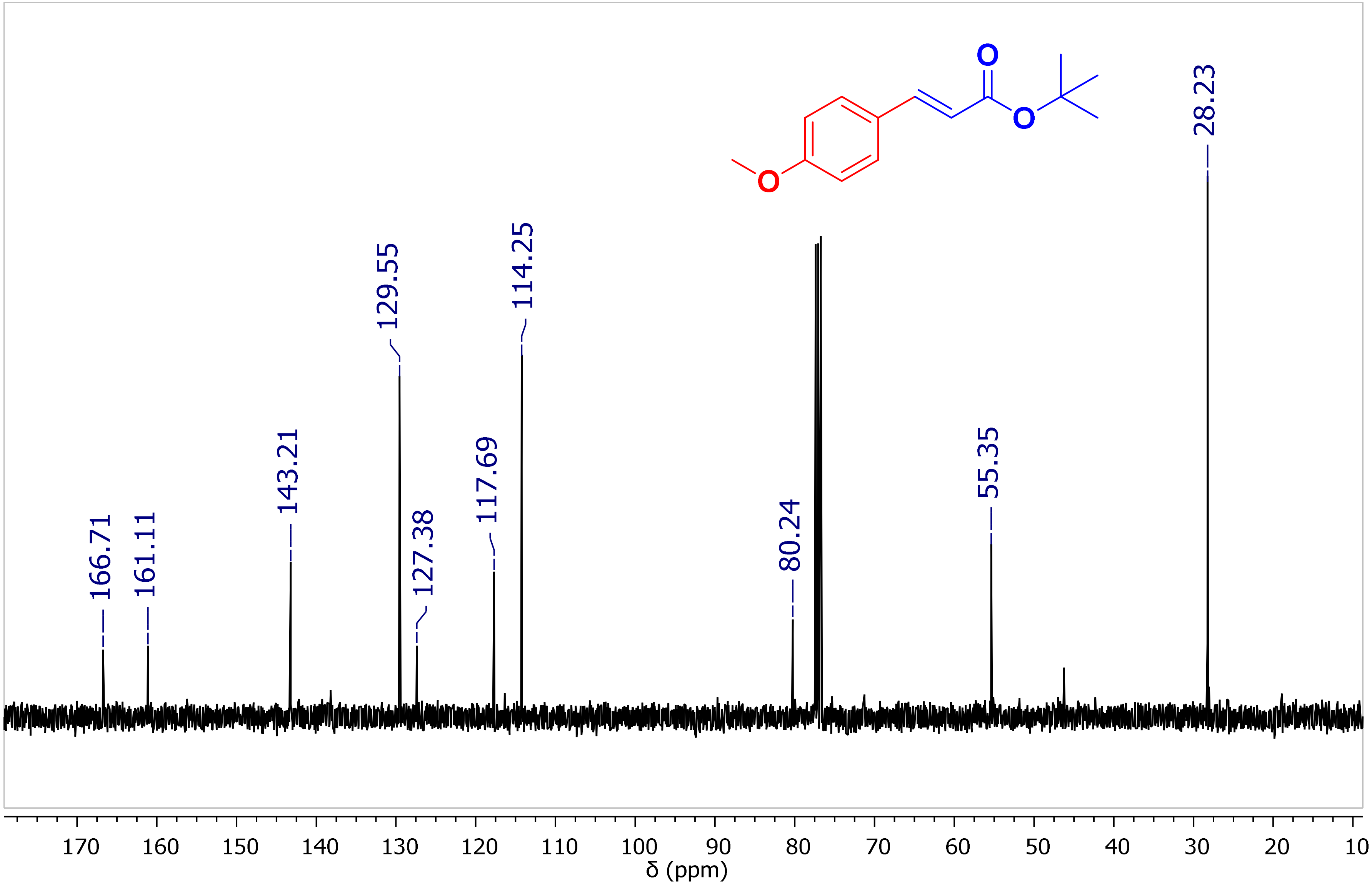

Figure S23. ^{13}C NMR (100 MHz, $CDCl_3$) of *tert*-butyl 4-methoxycinnamate. δ (ppm) 166.71, 161.11, 143.21, 129.55, 127.38, 117.69, 114.25, 80.24, 55.35, 28.23. The spectrum is consistent with previously reported data.[3]

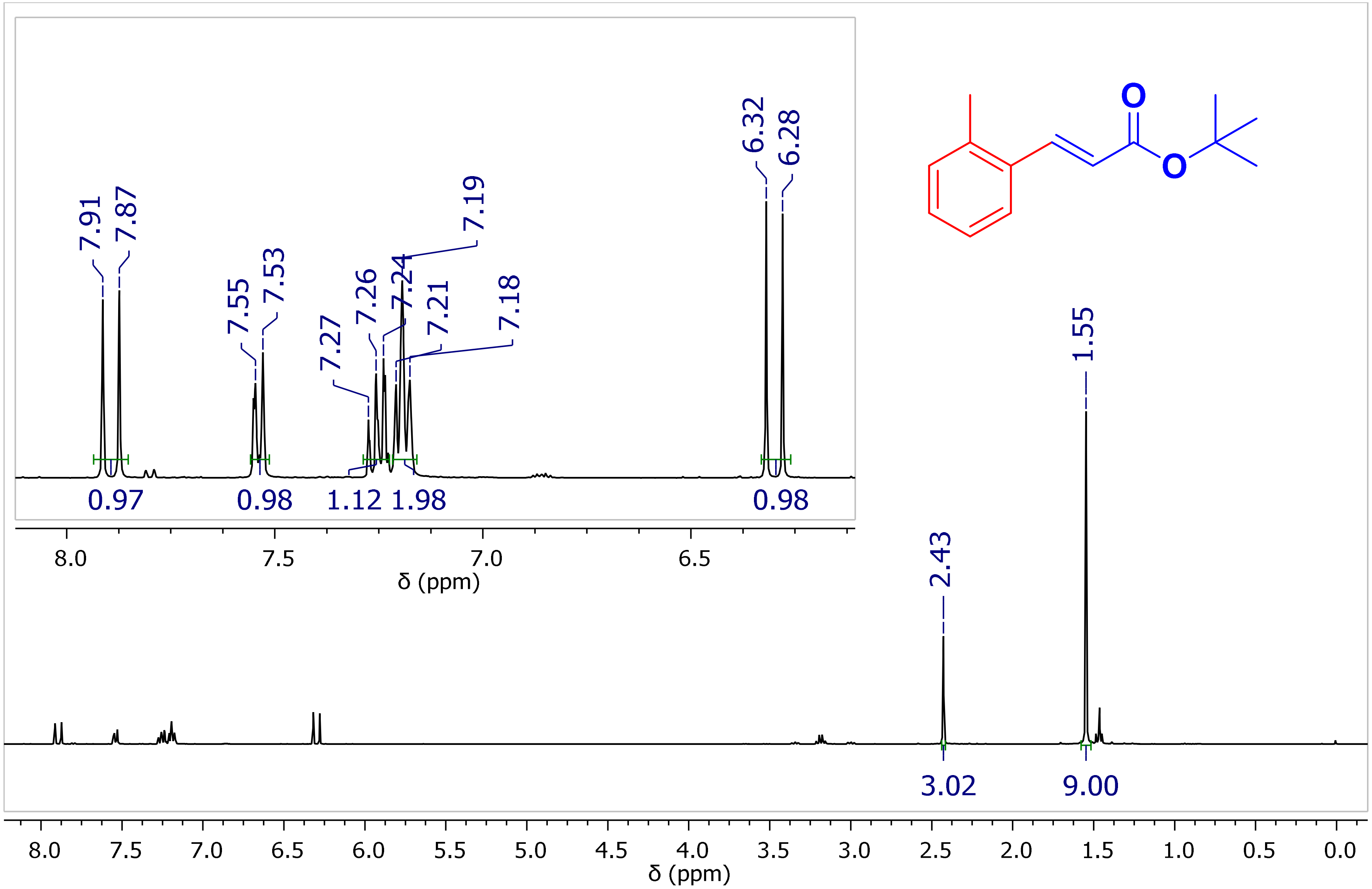

Figure S24. ^{1}H NMR (400 MHz, $CDCl_3$) of *tert*-butyl 2-methylcinnamate. δ (ppm) 7.91, 7.87, 7.55, 7.53, 7.27, 7.26, 7.24, 7.21, 7.19, 7.18, 6.32, 6.28, 2.43, 1.55. The spectrum is consistent with previously reported data.[5]

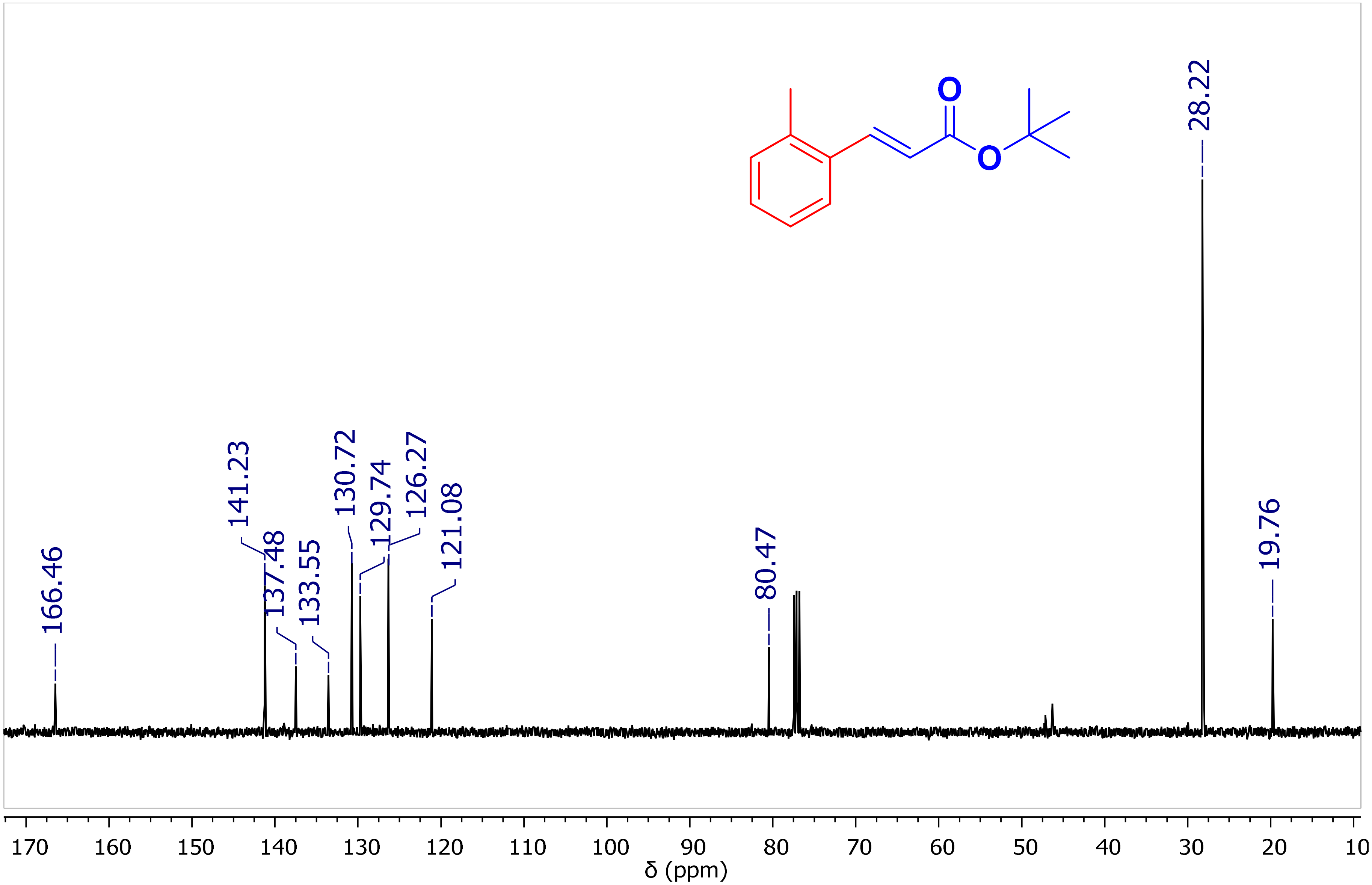

Figure S25. ^{13}C NMR (100 MHz, $CDCl_3$) of *tert*-butyl 2-methylcinnamate. δ (ppm) 166.46, 141.23, 137.48, 133.55, 130.72, 129.74, 126.27, 121.08, 80.47, 28.22, 19.76. The spectrum is consistent with previously reported data.[5]

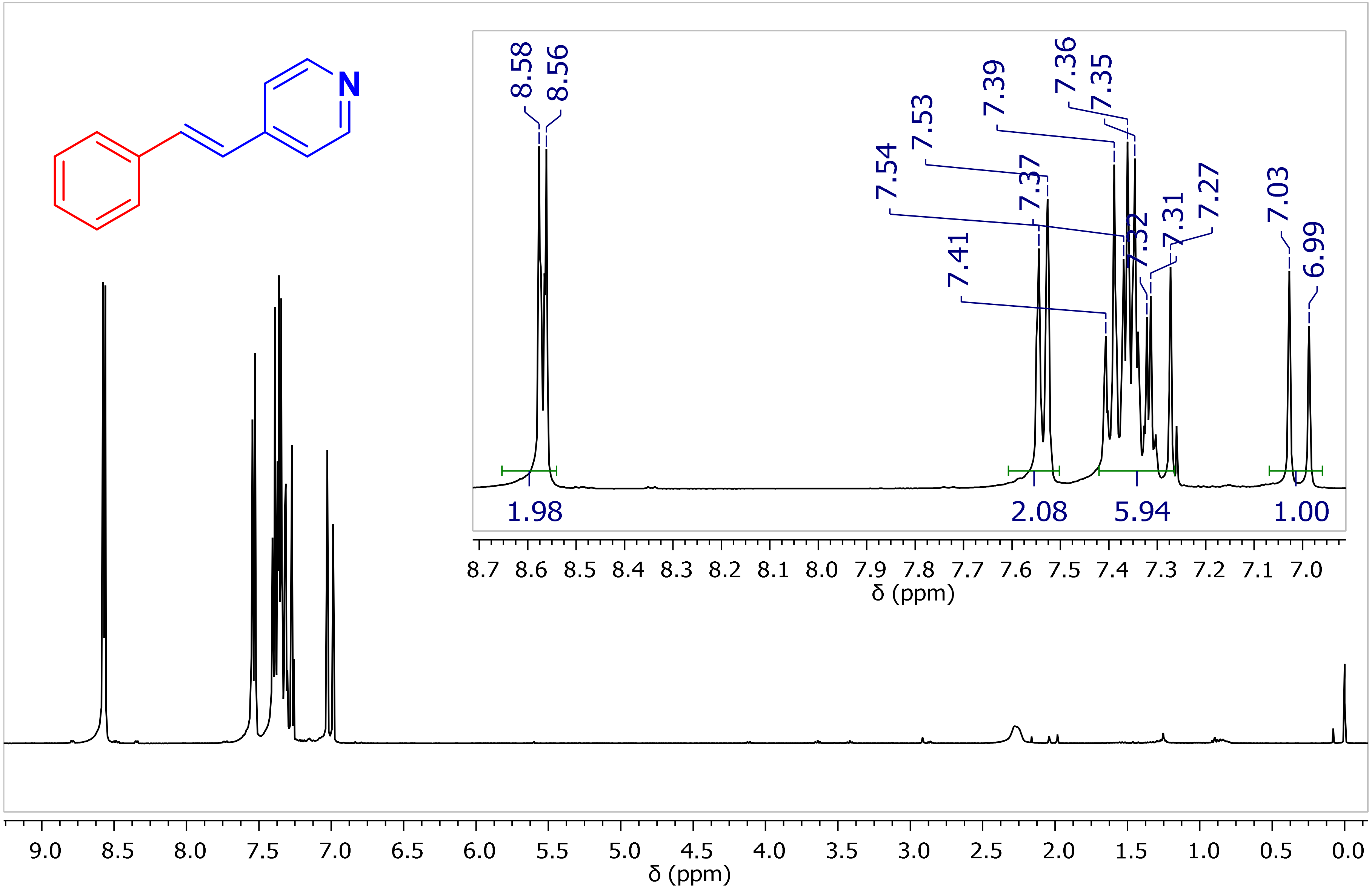

Figure S26. ^{1}H NMR (400 MHz, $CDCl_3$) of *trans*-4-styrylpyridine. δ (ppm) 8.58, 8.56, 7.54, 7.53, 7.41, 7.39, 7.37, 7.36, 7.35, 7.32, 7.31, 7.27, 7.03, 6.99. The spectrum is consistent with previously reported data.[6]

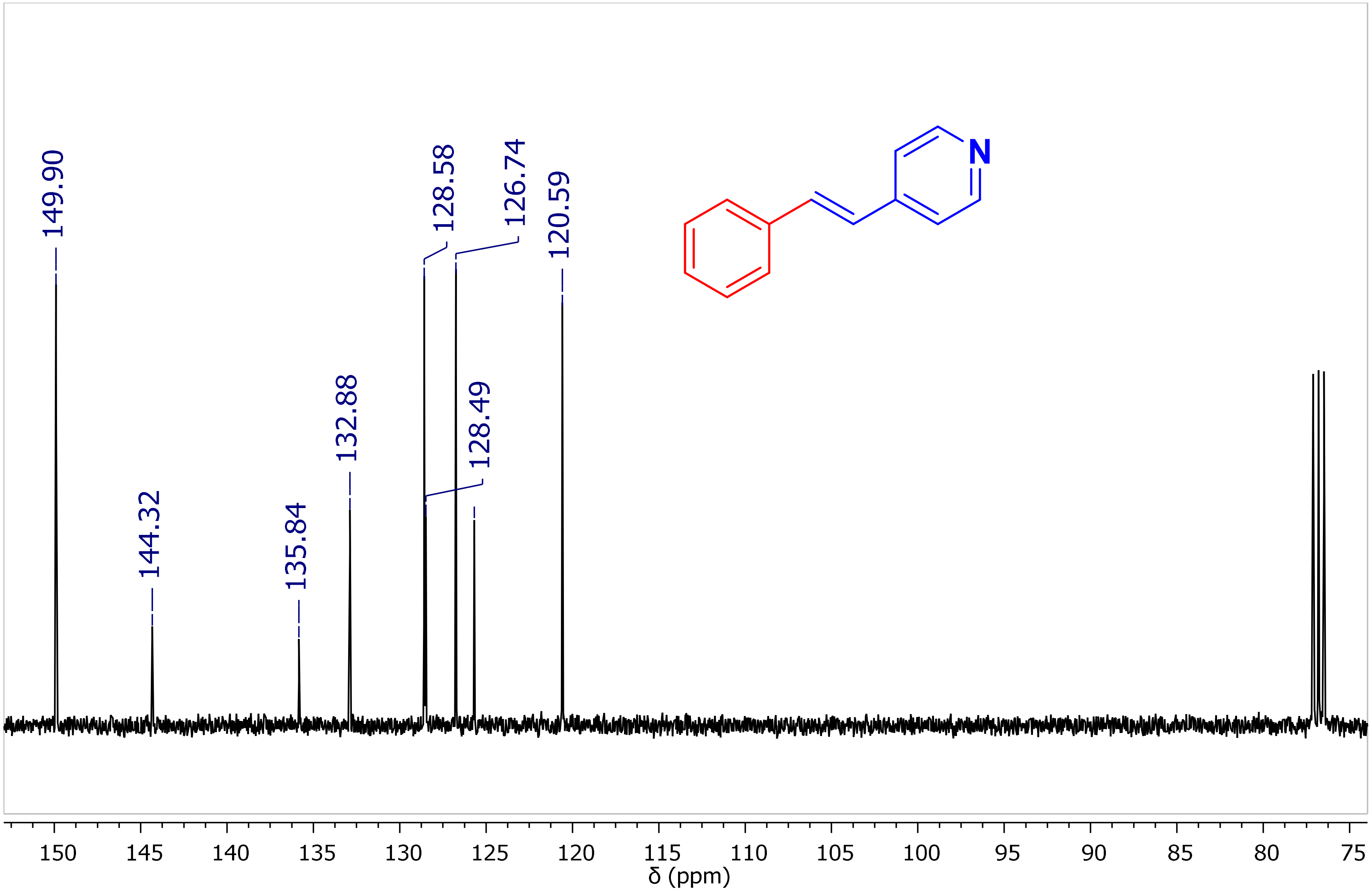

Figure S27. ^{13}C NMR (100 MHz, $CDCl_3$) of *trans*-4-styrylpyridine. δ (ppm) 149.90, 144.32, 135.84, 132.88, 128.58, 128.49, 126.74, 125.68,120.59. The spectrum is consistent with previously reported data.[6]

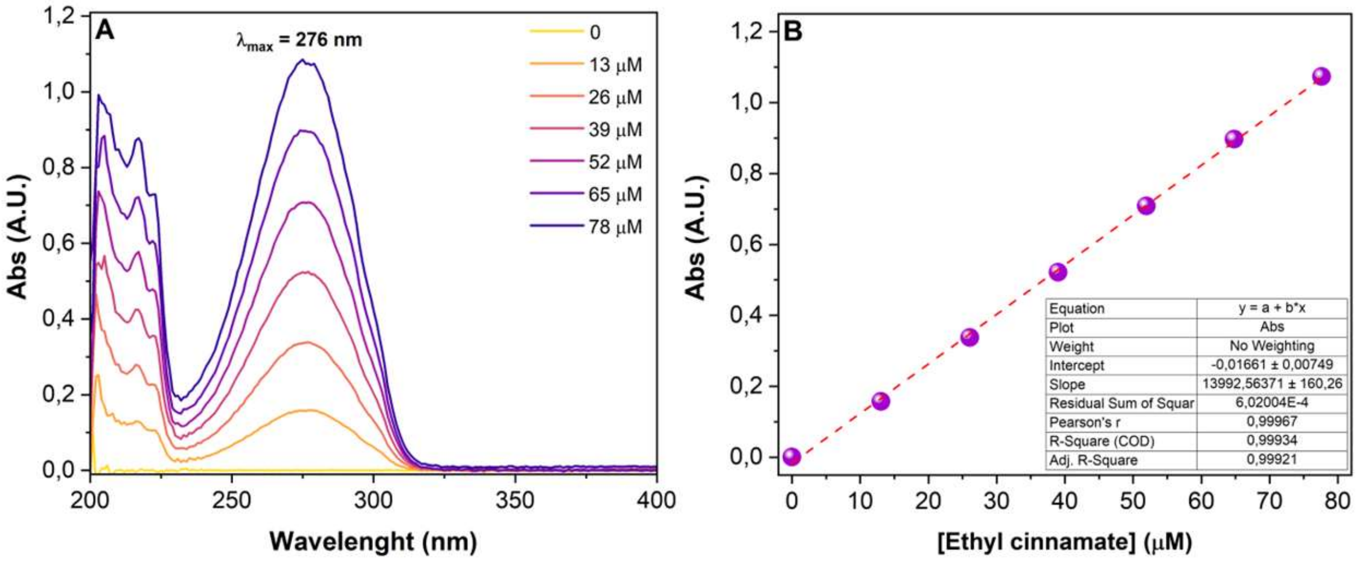

Figure S28. (a) Absorption spectra and (b) calibration curve for ethyl cinnamate at λ_{max} = 276 nm.

Powder X-ray diffraction (XRPD) data refinement description

During the Rietveld refinements, one linear and a 1/*x* background parameter, sample displacement, and metric parameters were optimized. The pseudo-Voigt profile function was deconvoluted into Gaussian and Lorentzian components, describing the average crystallite size (ACS as L_{Vol}-IB) and the micro-strain, respectively, for the Bragg reflection.

The average crystallite size and the crystallite size distribution (CSD) were further determined for the quasi-amorphous[7] polymer samples and the nano-crystalline Pd using the envelope function approach EnvACS.[8] The necessary pair distribuition functions (PDF) were calculated using pdfgetX3[9] from zero to 300 nm with steps of 3 pm of which the range between 0.1 nm and 280 nm could be used for the fitting of the envelope function, considering a MOACS[8] of 280 nm, and from 290 nm to 300 nm to compensate the Tuple analysis contribution of the Fourier riplle. Using Pawley fits for the background pattern observed using a measurement of an empty silicon waver and for the polymeric phases having variable and partially unknown chemical compositions, hypothetical symmetry and metric parameters were used to fit the respective reflection intensities for a full description of the observed scattering intensities.

The background of the instrument including Si waver sample holder was modeled using Pawley fits in space group *P*23 with a final lattice parameter of 1.7132 nm, an average crystallite size (ACS, calculated as L_{Vol}(IB)) of 1.09 nm and a scale factor of 1. The Pawley fit background function was set to zero and fixed during the refinements. After fitting the obtained intensities for the single reflections, they were as well fixed as the lattice parameter and the ACS. Thereafter, the scale factor was refined for reverse check and refined as sf_{bkg} = 0.962(39). The refinement results are plotted in **Figure S29**.

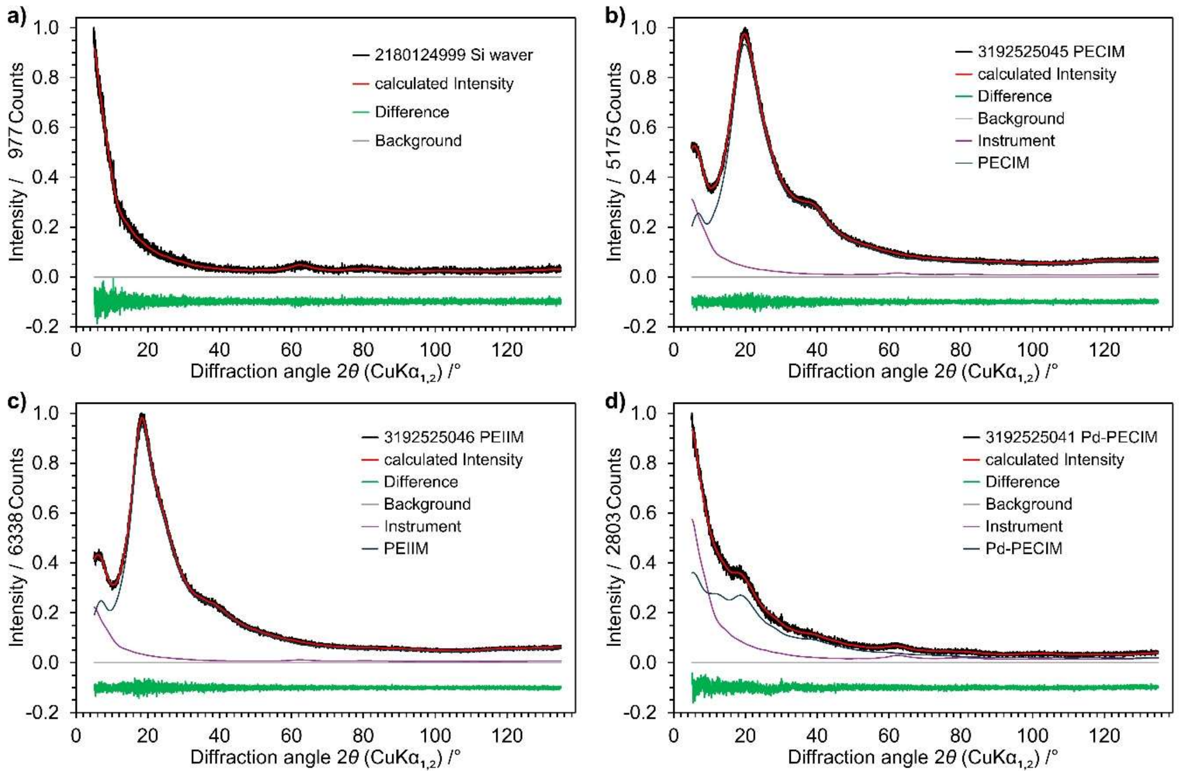

Figure S29. Pawley fit results for the collected X-ray powder diffraction data a) background measurement data using an empty silicon waver sample holder, b) the pure PECIM polymer, c) the pure PEIIM polymer, d) the Pd loaded PECIM polymer. The obtained intensities are given (black) together with the calculated sum of intensities (red) and the difference between both (green) as well as the background intensity used in the refinement, the instrumental background intensities determined from the Si waver measurement and the intensities of the polymer samples.

The calculated instrumental background data were used to further calculate in a second step a model for the different polymer samples. First, the data collected for PECIM (3192525045) were modeled using space group *P*222 with metric parameters of *a* = 0.44973 nm, b = 1.18467 nm, c = 1.50468 nm and V = 0.802(2) nm^9. After fitting the obtained intensities for the single reflections, they were fixed together with the lattice parameter and the calculated ACS = 1.4(2) nm. Thereafter, the scale factor was refined for reverse check and refined as sf_{bkg} = 1.01(4). Second, the data collected for PEIIM (3192525046) were modeled using the same space group (*P*222) and approximately the same lattice parameter as for PEIIM resulting in metric parameters of *a* = 0.44116 nm, b = 1.18863 nm, c = 1.52469 nm and V = 0.800(2) nm^9. After fitting the same procedure as for the instrumental background was used with an ACS = 1.2(2) nm. The scale factor reverse check gave sf_{bkg} = 1.00(4). Based on these obtained models the analysis of the freshly Pd loaded polymer (3192525041) was performed in a way that the scale factors for the instrumental background and the pure polymers were refined against the measured data. During this refinement it turned out that even combining all three models to describe the data obtained gave no satisfactory results. Therefore, the same *P*222 metric model used for the pure polymers was again refined using the above-described procedure. Metric parameters of *a* = 0.43960 nm, b = 1.17946 nm, c = 1.60286 nm and V = 0.831(2) nm^9 and an ACS = 0.8(2) nm were found.

For the samples running different number of Mizoroki–Heck reaction cycles the Bragg reflections of nano-crystalline Pd could be obtained. The Pd crystal structure was refined using the Rietveld method, including the model for the instrumental background and the different polymers. As none of the polymer models fit to the obtained amorphous scattering data, the same *P*222 model type was again used and refined with the Pawley method. The refinement results are summarized in **Table S1**.

To further validate not only the ACS but also the crystallite size distribution (CSD) the reciprocal space powder diffraction data were transferred to real space pair distribution function data[10,11] and the envelope function calculated without the need of a structure model using the EnvACS approach.[8] Within this approach the smallest and the broadest CSD of the used Howel function[12] was mixed with a broadening parameter (called ACS-PDF calculations[13]) to finally calculated the log-normal crystallite size maximum (LCS) and their distribution. Calculation result graphics are given in **Figure S30**.

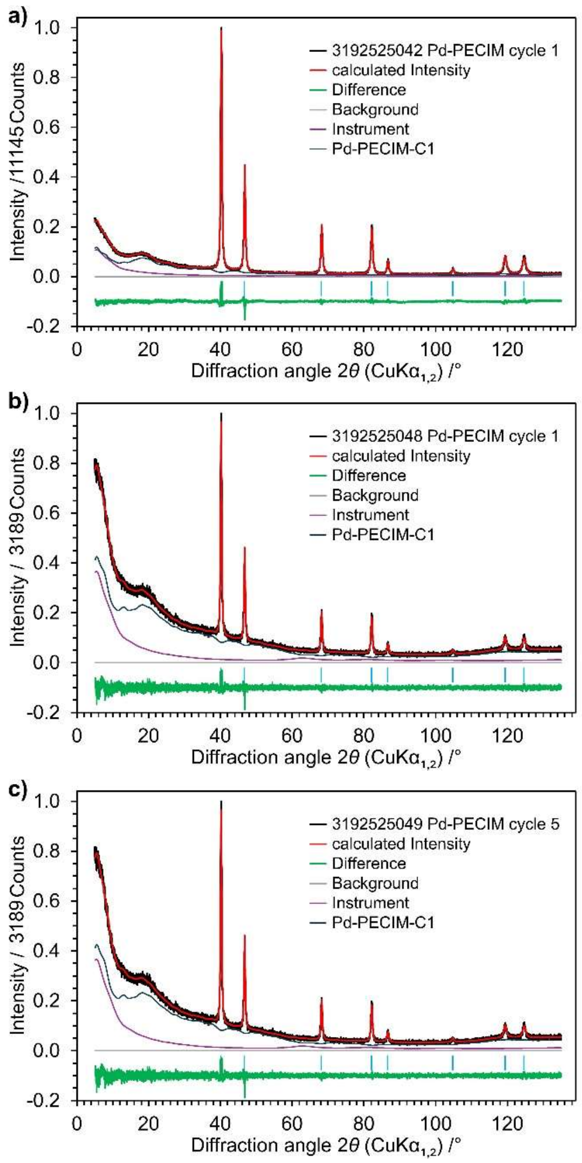

Figure S30. a) and b) Rietveld plot of two 1-times cycled samples, c) Rietveld plot of the 5-times cycled sample used also for b).

For the calculation of the ACS using the EnvACS approach a fitting range between 0.2 and 5 nm was used for the pure polymer samples as well as the freshly prepared Pd polymer showing only amorphous scattering. For the pure polymer samples within one standard deviation the same ACS is observed, with a very small CSD parameter. On the other hand, for the fresh Pd-PECIM sample (3192525041) the ACS is nearly 3-times bigger calculated (2.3(1) nm) than obtained from the Pawley fits (0.8(2) nm). At the same time the CSD is found to be very broad, as shown in **Figure S31**. This hints on one hand that the hypothetical unit cell selected seems to be too small (the bigger the unit cell the more reflections which will then be more narrow resulting in an increased ACS) and on the other hand to an uneven distribution of the Pd atoms linked to the polymer, which is consistent with the first assumption.

Analyzing the samples containing nano-crystalline Pd together with the amorphous scattering polymer it must be considered that the polymer contributes to the pair distribution function in the short distance range up to the approximated ACS of the polymer. Due to this effect data from distance greater than 2 nm were used (**Figure S31**). The maximum distance for the least-square minimization was set to 40 nm. Additionally, the scale factor was varied to account for the reduced $G^{norm}(r)_{max}$.

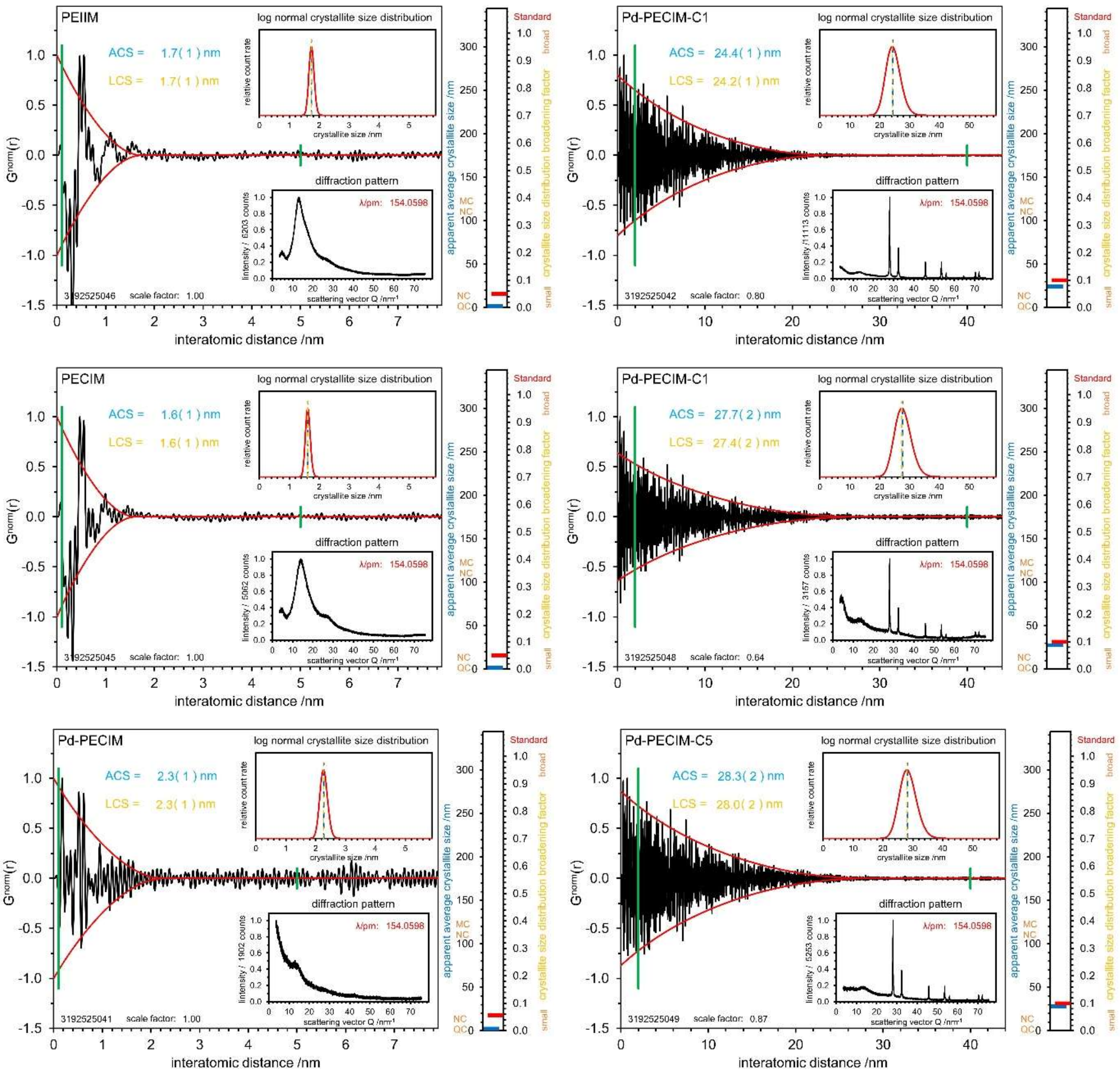

Figure S31. Determination of the average crystallite size (ACS) using the EnvACS approach[8] calculated as ACS-PDF. The crystallite size distribution (CSD) is given in the top inset, the background corrected diffraction pattern in the bottom inset. LCS corresponds to the crystallite size log-normal distribution maximum. Minimum and maximum PDF distances used are given with a long and short green vertical line, respectively.

Table S1. Unit cell volume (V_P) and average crystallite size (calculated as L_{Vol}(IB)) obtained for the polymer by Pawley fits (ACS_P), for palladium calculated by Rietveld refinements (ACS_R) and for both by EnvACS calculations (ACS_{PDF}). For the nano-crystalline Pd the $\mathbf{Fm\bar{3}m}$ lattice parameter (a) and micro-strain (ε_0) are as well given.

Sample ID	Sample USI[1]	V_P /nm^3	ACS_P /nm	ACS_{PDF} /nm	a_{Pd} /pm	ACS_R /nm	ε_0	ACS_{PDF} /nm
PEIIM	3192525046	0.800(2)	1.2(2)	1.7(1)				
PECIM	3192525045	0.802(2)	1.4(2)	1.6(1)				
Pd-PECIM	3192525041	0.831(2)	0.8(2)	2.3(1)				
Pd-PECIM C1*	3192525042	0.862(2)	1.7(2)	n.d.#	389.05(1)	15.9(1)	0.073(2)	24.4(1)
Pd-PECIM C1*	3192525048	0.864(2)	1.5(2)	n.d.#	389.07(1)	17.4(1)	0.059(3)	27.7(2)
Pd-PECIM C5*	3192525049	0.877(2)	1.4(3)	n.d.#	389.11(2)	18.1(9)	0.054(8)	28.3(2)

[1] USI: uniform sample identifier, * The number behind C represents the number of Mizoroki–Heck reaction cycles, # n.d. = not determined

References

(1) Strappaveccia, G.; Ismalaj, E.; Petrucci, C.; Lanari, D.; Marrocchi, A.; Drees, M.; Facchetti, A.; Vaccaro, L. A Biomass-Derived Safe Medium to Replace Toxic Dipolar Solvents and Access Cleaner Heck Coupling Reactions. *Green Chem.* **2015**, *17* (1), 365–372. https://doi.org/10.1039/C4GC01677G.

(2) Xu, W.; Liu, C.; Xiang, D.; Luo, Q.; Shu, Y.; Lin, H.; Hu, Y.; Zhang, Z.; Ouyang, Y. Palladium Catalyst Immobilized on Functionalized Microporous Organic Polymers for C–C Coupling Reactions. *RSC Adv.* **2019**, *9* (59), 34595–34600. https://doi.org/10.1039/C9RA07303E.

(3) Zhao, F.; Xin, L.; Zhang, Y.; Jia, X. Monodentate Phosphorus-Coordinated Palladium(II) Complexes as New Catalyst for Mizoroki-Heck Reaction of Aryl Halides with Electron-Deficient Olefins. *Chinese Chemical Letters* **2018**, *29* (3), 493–496. https://doi.org/10.1016/j.cclet.2017.08.004.

(4) Mohammadi, E.; Movassagh, B. Synthesis of Polystyrene-Supported Pd(II)-NHC Complex Derived from Theophylline as an Efficient and Reusable Heterogeneous Catalyst for the Heck-Matsuda Cross-Coupling Reaction. *Journal of Molecular Catalysis A: Chemical* **2016**, *418–419*, 158–167. https://doi.org/10.1016/j.molcata.2016.03.045.

(5) Bhunia, A.; Studer, A. Synthesis of Highly Substituted Arenes via Cyclohexadiene–Alkene C–H Cross Coupling and Aromatization. *ACS Catal.* **2018**, *8* (2), 1213–1217. https://doi.org/10.1021/acscatal.8b00083.

(6) Keesara, S.; Parvathaneni, S.; Mandapati, M. R. N,N′-Mono Substituted Acyclic Thioureas: Efficient Ligands for the Palladium Catalyzed Heck Reaction of Deactivated Aryl Bromides. *Tetrahedron Letters* **2014**, *55* (50), 6769–6772. https://doi.org/10.1016/j.tetlet.2014.09.053.

(7) Gesing, T. M.; Murshed, M. M.; Schuh, S.; Thüringer, O.; Krämer, K.; Neudecker, T.; Mendive, C. B.; Robben, L. Nano-Crystalline Precursor Formation, Stability, and Transformation to Mullite-Type Visible-Light Photocatalysts. *J. Mater. Sci.* **2022**, *57* (41), 19280–19299.

(8) Gesing, T. M.; Robben, L. Determination of the Average Crystallite Size and the Crystallite Size Distribution: The Envelope Function Approach EnvACS. *J. Appl. Crystallogr.* **2024**, *57* (5), 1466–1476.

(9) Juhás, P.; Davis, T.; Farrow, C. L.; Billinge, S. J. L. *PDFgetX3*: A Rapid and Highly Automatable Program for Processing Powder Diffraction Data into Total Scattering Pair Distribution Functions. *J. Appl. Crystallogr.* **2013**, *46* (2), 560–566.

(10) Egami, T.; Billinge, S. J. L. Underneath the Bragg Peaks. *Materials Today* **2003**, *6* (6), 57.

(11) Billinge, S. J. L. The Rise of the X-Ray Atomic Pair Distribution Function Method: A Series of Fortunate Events. *Phil. Trans. R. Soc. A.* **2019**, *377* (2147), 20180413.

(12) Howell, R. C.; Proffen, T.; Conradson, S. D. Pair Distribution Function and Structure Factor of Spherical Particles. *Phys. Rev. B* **2006**, *73* (9), 094107.

(13) Jundullah Hanafi, Md. I.; Murshed, M. M.; Robben, L.; Gesing, T. M. Plagioclase Feldspars $(Ca_{1-x}Na_x)(Al_{2-x}Si_{2+x})O_8$: Synthesis and Characterizations of Mechanical Weathering Relevant to Martian Regolith. *Zeitschrift für Kristallographie - Crystalline Materials* **2025**, *240* (1–2), 13–27.
